# The Effect of Acute Beetroot Juice and Caffeine Supplementation on Specific Judo Performance During a Simulated Competition Day in Judo Athletes

**DOI:** 10.1002/ejsc.70278

**Published:** 2026-10-21

**Authors:** Sefer Karabağ, Heidar Sajedi, Onur Güler, Vahdet Alaeddinoğlu, Ergün Çakır, Davar Khodadadi, Selahattin Akpınar

**Affiliations:** ^1^ Sarıkamış Anatolian Imam Hatip High School Ministry of National Education Sarıkamış Kars Turkey; ^2^ Department of Movement and Training Sciences Sport Science Faculty Duzce University Duzce Turkey; ^3^ Department of Exercise and Sports Science for Disabled People Faculty of Health Science International Science and Technology University Warsaw Poland; ^4^ Department of Sports Health Sciences Graduate School of Winter Sports and Sports Sciences Ataturk University Erzurum Turkey; ^5^ Department of Reaction Sciences Faculty of Sports Sciences Ataturk University Erzurum Turkey; ^6^ Department of Coaching Education Faculty of Sports Sciences Van Yuzuncu Yıl University Van Turkey; ^7^ Faculty of Physical Education and Sports Sciences Islamic Azad University Central Tehran Branch Tehran Iran

**Keywords:** beetroot juice, caffeine, cognitive function, muscle oxygenation, neuromuscular function, simulated judo competition

## Abstract

Beetroot juice (BJ) and caffeine (CAF) are well‐established ergogenic aids for exercise performance. However, little is known about their efficacy and interaction within the specific context of a judo competition day. This study investigated the acute effects of BJ, CAF, and their combination (BJCAF) on judo‐specific performance across a simulated competition day. Using a randomized, double‐blind, placebo‐controlled crossover design, 35 competitive judo athletes (age 23 ± 5 years; body mass 74 ± 13 kg) completed 4 sessions (BJ, CAF, BJCAF, and placebo), each consisting of 5 trials of judo randori and a Special Judo Fitness Test (SJFT), followed by neuromuscular and cognitive assessments. Compared with placebo, CAF enhanced SJFT performance, neuromuscular function, and cognition in trials 1–3 but not in trial 5 (*p* < 0.05). Conversely, BJ improved these same metrics in trial 5 (*p* < 0.05). BJCAF enhanced SJFT performance across trials 1–3 and 5, cognitive functions in trials 1, 2, and 4, and neuromuscular performance in all trials (*p* < 0.05). Importantly, in trial 5, both BJ and BJCAF conditions yielded improved SJFT performance, neuromuscular function, and cognitive outcomes compared to CAF alone (*p* < 0.05). The results demonstrate that the ergogenic effect of CAF was not maintained throughout the simulated competition day, whereas BJ provided late‐stage benefits. Co‐ingestion effectively counteracted the later‐session decline observed with CAF alone while maintaining its early advantages. This highlights the importance of considering temporal dynamics and metabolic costs when designing nutritional strategies for multi‐bout, high‐intensity sports tournaments such as judo.

## Highlights

- Ergogenic effects of caffeine supplementation are primarily short‐lived and are followed by a marked decline in judo‐specific performance, neuromuscular function, and cognitive vigilance under simulated competition conditions.
- Beetroot juice produced a late‐stage benefit, improving exercise efficiency and supporting cardiovascular recovery during later bouts.
- Combined caffeine and beetroot juice supplementation produced the most favorable overall profile, attenuating the late performance decrement observed with caffeine alone and supporting sustained high‐level performance across the competition day.

## 1 Introduction

Judo is a high‐intensity, intermittent grappling sport that demands advanced technical skill and a complex mix of muscle power, anaerobic power, aerobic power, aerobic capacity, muscular strength, strength‐endurance, and tactical decision‐making (Miarka et al. 2012). A typical competition day in judo tournaments, which often comprises five to seven matches, imposes substantial physiological and cognitive demands, since athletes must sustain peak performance across multiple bouts with limited recovery (Detanico et al. 2015; Julio et al. 2017). This competition structure promotes progressive central and peripheral fatigue, which commonly manifests as reduced muscle power, impaired vigilance, and altered metabolic function, all of which can decisively influence competitive outcomes (Campos et al. 2025; Detanico et al. 2015; Serrano‐Huete et al. 2016; Stavrinou et al. 2016). Consequently, efficient recovery and the capacity to maintain performance over successive matches are critical for judo athletes, and even marginal gains can determine medal placement (Franchini et al. 2009). Athletes, therefore, frequently turn to targeted, evidence‐based nutritional strategies; however, consensus guidelines note that only a limited number of supplements have sufficient scientific support for performance enhancement. Among these, caffeine (CAF) and dietary nitrate, typically consumed as beetroot juice (BJ), are recognized as evidence‐based ergogenic aids with demonstrated efficacy across a range of exercise modalities (Burke 2017).

CAF (1,3,7‐trimethylxanthine) is a central nervous system stimulant present in coffee, tea, cacao, and many sports supplements. Because it is structurally similar to adenosine, CAF antagonizes A_1_ and A_2A_ receptors, thereby reducing adenosine‐mediated inhibition and increasing central drive (Sajedi et al. 2025). This action elevates arousal and alertness, lowers perceived exertion at a given workload, and facilitates neuromuscular recruitment (Davis and Green 2009; Guest et al. 2021). Peripherally, CAF increases circulating catecholamines and heart rate (HR), which can stimulate glycolysis and increase the substrate availability for working muscle (Laurent et al. 2000). At the muscle level, CAF appears to enhance excitation–contraction coupling, partly via increased sarcoplasmic reticulum calcium release, and may augment motor‐unit firing rate, together supporting greater peak power output (Davis and Green 2009). These central and peripheral actions translate into consistent performance benefits across sport‐specific tasks, including improved intermittent‐sprint performance, strength and power outputs, and endurance (Astorino and Roberson 2010; Grgic et al. 2018; Khodadadi et al. 2025). CAF also reliably improves simple cognitive functions such as attention and reaction speed (Lorenzo Calvo et al. 2021), an effect that is especially relevant in fast‐paced combat sports. In these sports, acute CAF ingestion has been shown to enhance sport‐specific metrics, such as increasing the number of throws in judo (Durkalec‐Michalski et al. 2019), kick frequency in taekwondo (Ouergui et al. 2023), and mean power in simulated boxing bouts (Coswig et al. 2018). However, not all findings are positive; for instance, Filip‐Stachnik et al. (2021) reported that caffeinated chewing gum (5.4 mg/kg) did not enhance performance in repeated Special Judo Fitness Tests (SJFT).

Dietary nitrate, most commonly administered as concentrated BJ, provides an alternative ergogenic pathway. Following ingestion, nitrate is sequentially reduced to nitrite and then to nitric oxide (NO), a process that is accelerated under hypoxic and acidic conditions. Nitric oxide induces vasodilation and modulates muscle contractile efficiency (Stamler and Meissner 2001). Supplemental studies have demonstrated that dietary nitrate can reduce the oxygen cost of submaximal exercise, enhance muscle perfusion and mitochondrial efficiency, and accelerate phosphocreatine resynthesis between exercise bouts (Clements et al. 2014; Larsen et al. 2007; Vanhatalo et al. 2011). Collectively, these adaptations are thought to support repeated high‐intensity efforts by delaying fatigue and reducing reliance on anaerobic pathways. In support of this notion, Thompson et al. (2015) reported that 7 days of nitrate supplementation increased total sprint work and attenuated the decline in cognitive function (reaction time, RT) during a 2 × 40 minutes intermittent sprint protocol. Beyond peripheral mechanisms, dietary nitrate may also exert central effects; in certain exercise tasks, it has been shown to improve mood and reduce ratings of perceived exertion (RPE) at equivalent workloads, potentially through attenuation of group III–IV muscle afferent feedback associated with fatigue (Husmann et al. 2019). Overall, nitrate supplementation has demonstrated ergogenic benefits primarily in endurance and repeated‐sprint exercise, often accompanied by favorable cognitive and perceptual responses.

Despite the well‐established ergogenic potential of CAF and dietary nitrate when administered individually, little is known about their efficacy and interaction within the specific context of a judo competition day. The isolated effects of each supplement have been only sparsely investigated in judo and related combat sports. For example, Demirli et al. (2025) reported that acute BJ supplementation increased jump height, back muscle strength, handgrip strength (HGS), and total number of throws (TT) during the SJFT in adolescent judo athletes. Guler et al. (2026) indicated that BJ primarily attenuated the physiological cost of performance and accelerated recovery during the SJFT, rather than directly increasing performance. Similarly, Carmo et al. (2021) demonstrated that ingestion of 5 mg/kg CAF improved post‐training SJFT throws and reduced the fatigue index in adult judo athletes during a heavy training session. However, these investigations focused on single bouts or training protocols rather than the cumulative demands of multiple competitive matches. The physiological stress imposed by a single maximal effort differs substantially from the progressive fatigue that develops across a tournament day. Only a limited number of studies have examined CAF supplementation during repeated‐bout exercise. While some report that CAF's ergogenic effects are maintained across successive bouts (Hornery et al. 2007), others suggest a diminished or even detrimental effect during later exercise periods, potentially arising from greater disruption of homeostasis (e.g., greater glycogen utilization, increased metabolite accumulation) or a rebound effect following initial stimulation (Crowe et al. 2006; Greer et al. 1998). Collectively, these contradictory findings underscore a critical unresolved question: can CAF's performance‐enhancing effects be sustained across repeated matches, or does its early benefit precipitate a premature performance decline in later stages of competition?

Similarly, the role of BJ within a repeated high‐intensity competition model remains unclear. Its primary mechanism—improving metabolic efficiency—may be particularly advantageous during later matches by preserving muscle function and delaying fatigue (Alsharif et al. 2023). Notably, the physiological effects of CAF, such as increased adrenergic activity and vasoconstriction, and those of BJ, including NO‐mediated vasodilation and enhanced muscle perfusion, are in several respects opposing. This contrast raises a compelling and largely unexplored question: can the vasodilatory and efficiency‐enhancing properties of BJ offset potential late‐tournament performance declines associated with CAF, or can their combined ingestion sustain physical output and cognitive performance across successive matches? To date, research examining the combined use of CAF and BJ in sport is limited and yields mixed findings, ranging from additive ergogenic effects (Felippe et al. 2016; Liu et al. 2025; Montalvo‐Alonso et al. 2025) to no additional benefit beyond CAF alone (Glaister et al. 2015; Lane et al. 2014; Pollex et al. 2025). Importantly, no study has yet investigated this interaction within the ecologically valid context of a simulated judo competition day.

Therefore, the primary aim of this study was to examine the acute effects of CAF, BJ, and their combined ingestion on sport‐specific performance, physiological responses, and cognitive function across a series of trials designed to replicate the demands of a judo tournament in competitive judo athletes. We hypothesized that the ergogenic effects of CAF on judo‐specific performance would not be sustained across the final trials and that its early benefits might contribute to a subsequent performance decline, whereas BJ co‐ingestion would attenuate this decline.

## 2 Methods

### 2.1 Study Design

This study used a randomized, double‐blind, placebo‐controlled crossover design. Each athlete completed four identical experimental sessions separated by at least a 10‐day washout period. Conditions were BJ, CAF, BJ + CAF (BJCAF), and placebo (PLA). To control for potential order effects, supplementation conditions were administered according to a counterbalanced Latin‐square design. This approach ensured that each condition appeared equally often in each testing session and that treatment order was balanced across participants. Sequence allocation was generated by a computer and implemented by an independent researcher who was not involved in data collection.

### 2.2 Participants

Thirty‐five competitive judo athletes (23 males and 12 females; age, 23 ± 5 years; body mass, 74 ± 13 kg; height, 177 ± 8 cm; maximal oxygen uptake (${\dot{V}O}_{2}$max), 52.68 ± 5.65 ml/kg/min; mean ± SD) were recruited from the initial 47 volunteers. Twelve individuals were excluded because of habitual CAF consumption > 1 mg/kg/day (*n* = 5), musculoskeletal injury within the preceding 3 months (*n* = 3), current use of nutritional supplements (*n* = 3), or pregnancy (*n* = 1). The CONSORT flow diagram is shown in Figure 1. Eligibility was assessed via medical screening and a detailed questionnaire. Inclusion criteria were age 18–30 years; ≥ 3 years of competitive judo experience and current training ≥ 3 sessions/week, habitually low CAF consumption (≤ 0.99 mg/kg/day) (Filip et al. 2020), no musculoskeletal injury in the preceding 3 months, and no smoking or alcohol use. Exclusion criteria were: any illness, injury or condition limiting maximal performance (Sajedi et al. 2025); any sign of CAF‐withdrawal symptoms (e.g., headache, fatigue, reduced energy or alertness, drowsiness, depressed mood, difficulty concentrating, irritability, yawning (Juliano and Griffiths 2004)) during the initial free‐CAF month; known intolerance or hypersensitivity to CAF or nitrate‐rich supplements; use of medications, supplements, or ergogenic aids affecting nitric oxide (NO) or CAF metabolism (e.g., nitrates, nitrites, NO donors, PDE‐5 inhibitors, *β*‐blockers, stimulants); CAF consumption > 25 mg/day for > 3 days during the study; vastus lateralis skinfold > 15 mm; inability to complete experimental trials; or non‐adherence to study recommendations. Female athletes were tested during the late follicular phase of the menstrual cycle. All participants provided written informed consent. The study was approved by the Ethics Committee of International Science and Technology University (202507‐01) and conducted in accordance with the Declaration of Helsinki. The trial was registered with the Iranian Registry of Clinical Trials (IRCT20231004059615N5).

**FIGURE 1:**
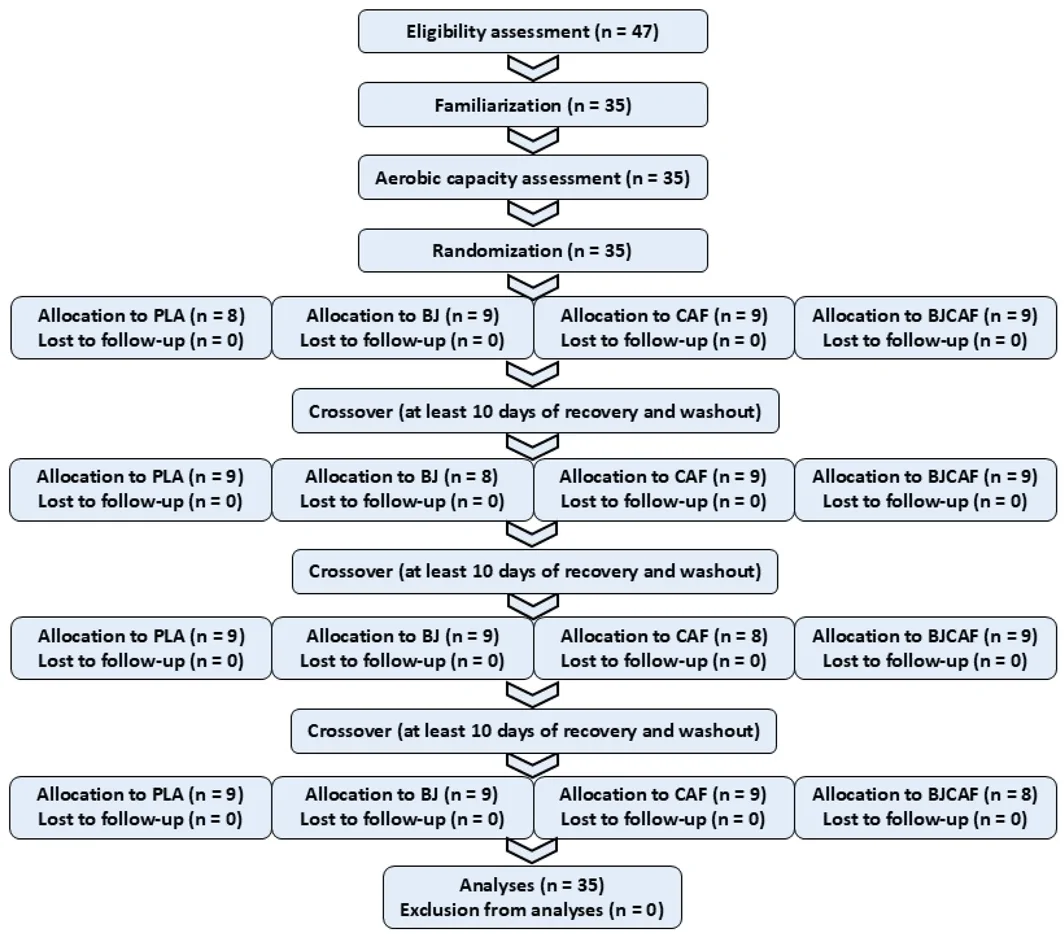
The CONSORT flow diagram.

### 2.3 Procedures

A priori power analysis was performed using G*Power v3.1.9.4. We selected F tests‐analysis of variance (ANOVA): repeated measures, within factors, with the following inputs: effect size *f* = 0.25, *α* = 0.05, power = 0.80, number of groups = 1, number of measurements = 4, correlation among repeated measures = 0.50, nonsphericity correction *ε* = 1.0. The required total sample size was *n* = 24. However, to account for dropouts and to increase the power for secondary analyses, 35 participants were recruited.

Prior to the experiments, volunteers attended familiarization and ${\dot{V}O}_{2}$max testing sessions separated by 5 days. The familiarization session replicated the tests, timing, and sequences used in the experimental sessions, except that only two trials were performed. Although participants were classified as low CAF consumers, they were asked to reduce CAF intake to < 25 mg/day for 1 month before the experiments to minimize habituation/withdrawal effects (Khodadadi et al. 2025). Participants were also instructed to avoid nitrate‐containing foods and supplements for 2 weeks prior to testing. Daily food and fluid intake were logged using *MyFitnessPal*, and adherence was monitored. The pretrial diet was standardized for the 24 h before familiarization and each experimental session (high carbohydrate: ≈ 70% carbohydrate, 15% protein, 15% fat). Participants avoided antibacterial mouthwash and strenuous exercise for 48 h before each session. To minimize the potential influence of circadian variation (Aydin et al. 2026), participants attended all experimental sessions at the same time of day, with a standardized breakfast provided at 07:00. Verbal encouragement was standardized across sessions. The familiarization and experimental sessions were conducted in the same judo gym (temperature 21.5 ± 0.6°C; relative humidity 30 ± 10%).

### 2.4 Aerobic Power Assessment

After a 10‐minute standardized warm‐up, ${\dot{V}O}_{2}$max was determined using a Bruce incremental protocol on a motorized treadmill (Varilant, Lode, The Netherlands) in a climate‐controlled laboratory. The test began at 2.7 km/h with a 10% incline and increased in speed and grade every 3 minutes until volitional exhaustion (inability to maintain workload despite encouragement). Breath‐by‐breath ${\dot{V}O}_{2}$ was recorded with a calibrated metabolic system (PowerCube‐Ergo, Ganshorn Medizin Electronic GmbH, Germany). Achievement of ${\dot{V}O}_{2}$max required at least two of the following: ${\dot{V}O}_{2}$ plateau, HR ≥ 90% of age‐predicted HRmax, respiratory exchange ratio (RER) > 1.20, or RPE ≥ 18 (Borg scale). The age‐predicted HRmax was calculated using the equation HRmax = 208 − (0.7 × age) proposed by Tanaka et al. (2001). A 5‐minute cool‐down (3 km/h, 0% incline) followed the test. ${\dot{V}O}_{2}$ was defined as the highest 10‐second ${\dot{V}O}_{2}$ value (Siahkouhian et al. 2013).

### 2.5 Experimental Session Structure

Participants arrived at the gym at 07:00 and, after breakfast, rested in a lying or seated position. Following a 15‐minute warm‐up (10 minutes general, 5 minutes judo specific), participants completed five identical trials separated by controlled recovery intervals (20 minutes) to mimic a quasi‐competition day. Each trial comprised a 4‐minute high‐intensity judo randori immediately followed by the Special Judo Fitness Test (SJFT), with a 30‐second interval for judogi arrangement. Assessments of neuromuscular function (Countermovement jump (CMJ) and maximal isometric HGS) and cognitive performance were conducted pre‐exercise (before trial 1) and after each trial, with 30 seconds between consecutive assessments. Between trials, participants performed a 5‐minute active recovery (jogging/walking), followed by 10 minutes of seated rest and a 5‐minute re‐warm‐up. Twenty‐five minutes after the final trial, participants completed an additional SJFT during which muscle oxygenation was recorded. All tests were administered at the same time points and in the same order across sessions to avoid order effects. Sessions ended with a 15‐minute cool‐down. The first trial began at 09:00, and the total session lasted approximately 5.5 hours (07:00–12:30). Immediately after each trial, participants consumed a standardized isotonic drink (Gatorade Endurance Formula) at 3 mL/kg to support hydration and energy availability; drinks were premeasured, chilled (10°C–12°C), and consumed under supervision within 2 minutes. No additional fluids, caloric beverages, or snacks were permitted. The experimental session structure is illustrated in Figure 2.

**FIGURE 2:**
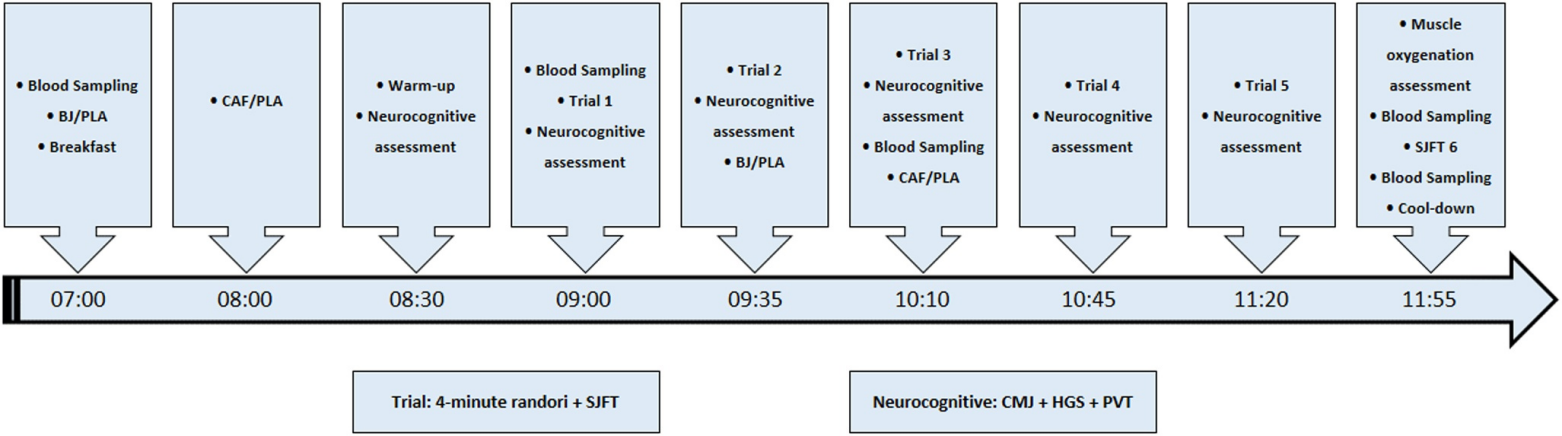
The structure of the experimental session. BJ, beetroot juice; BJCAF, beetroot juice + caffeine; CAF, caffeine; CMJ, countermovement jump; HGS, handgrip strength; PLA, Placebo; PVT, Psychomotor Vigilance Test; SJFT, Special Judo Fitness Test.

### 2.6 Judo Randori

Each trial began with a 4‐minute high‐intensity randori performed against an opponent matched for body mass (± 3 kg) and skill level. Participants faced the same opponents in each session; however, the order of opponents within each trial was randomized. Randori emphasized continuous engagement rather than scoring and was supervised by an official judo referee. Verbal encouragement was standardized, and participants were instructed to maintain maximal effort throughout the bout.

The 4‐minute randori protocol was selected because it closely replicates the duration and physiological demands of official judo competition. Previous research has demonstrated good‐to‐very‐large test–retest reliability for several time‐motion and physiological responses during simulated judo matches, supporting the reproducibility of this type of protocol (Franchini et al. 2020).

### 2.7 Special Judo Fitness Test (SJFT)

The SJFT is a judo‐specific intermittent field test that reproduces the repeated throwing efforts and brief recovery periods characteristic of judo combat, making it a practical proxy for selected high‐intensity technical and physical demands of judo combat (Sterkowicz‐Przybycień et al. 2019). The test consists of three work periods (15, 30, and 30 seconds) separated by 10‐second recovery intervals, during which participants perform as many *ippon‐seoi‐nage* throws as possible against two opponents. The total number of throws (TT) was recorded, and heart rate was continuously monitored using a Polar H10 monitor (Polar Electro Oy, Finland). The SJFT index was calculated as (HR immediately posttest [HR_0_] + HR 1 minute posttest [HR_1_])/TT, with lower values indicating better performance. Previous studies have reported good reliability for the SJFT index (ICC = 0.81), although reliability varies among individual SJFT outcomes (Štefanovský et al. 2021). RPE (Borg 6–20 scale) was recorded immediately after completion of the final work period (the third execution period).

### 2.8 Countermovement Jump (CMJ)

CMJ performance was assessed to evaluate lower‐body power and neuromuscular function. Participants performed two maximal CMJs at each time point with their hands placed on their hips to eliminate arm swing. Trials were separated by 30 seconds of passive recovery, and the best performance was retained for analysis. Jumps were performed on a force platform (400 Series, Fitness Technology, Australia), sampling at 1000 Hz. Flight time was determined from the force–time curve as the interval between take‐off and landing, and jump height was calculated using the flight‐time method (*h* = gt^2^/8, where *g* = 9.81 m·s^−2^). The flight‐time‐to‐contraction‐time (FT:CT) ratio was calculated as flight time divided by contraction time (from movement onset to take‐off) (Cormack et al. 2008). Participants performed all jumps barefoot, and testing instructions and data‐processing procedures were standardized across sessions. Previous research in judo athletes has reported excellent reliability for CMJ‐derived variables (ICC = 0.90–0.99) (Chang et al. 2022).

### 2.9 Handgrip Strength (HGS)

Maximal HGS was assessed using a digital dynamometer (Danesh Salar Iranian, Iran). Participants performed two maximal 3‐second contractions with their dominant hand, separated by 30 seconds of passive recovery. The highest value (kg) was used for analysis. Previous studies in judo athletes have reported good‐to‐excellent reliability for handgrip strength assessment, with ICC values exceeding 0.85 (Kons et al. 2021).

### 2.10 Psychomotor Vigilance Test (PVT)

Cognitive performance was assessed using a 3‐minute PVT administered on a laptop computer in the judo gym under standardized lighting and ambient noise conditions while participants remained seated. A red visual stimulus appeared at random interstimulus intervals ranging from 1 to 5 seconds, and participants were instructed to respond as quickly as possible by pressing the space bar with their preferred finger. Outcome measures included the number of lapses (RT ≥ 355 milliseconds) and response speed (mean 1/RT). Reaction times ≤ 130 milliseconds were classified as anticipatory responses and excluded from analysis (Benderoth et al. 2021). The pre‐exercise PVT lasted 4 minutes, with the first minute serving as familiarization and excluded from subsequent analyses. Previous studies have reported good reliability for the 3‐minute PVT (ICC ≈ 0.80) (Smith et al. 2021).

### 2.11 Muscle Oxygenation

Muscle oxygenation was recorded continuously during the final SJFT using near‐infrared spectroscopy (NIRS; Portamon, Artinis Medical Systems, The Netherlands). The portable probe was placed on the dominant vastus lateralis and secured with adhesive tape and an opaque cover to minimize motion and ambient‐light artifacts. Data were sampled at 10 Hz. The tissue saturation index (TSI) was calculated by the manufacturer's software from changes in oxygenated hemoglobin (O_2_Hb), deoxygenated hemoglobin (HHb), and total hemoglobin (tHb). Baseline TSI was the mean over the last 30 seconds of a 5‐min seated rest preceding the SJFT. TSI was analyzed separately for each SJFT bout and inter‐bout recovery. Skinfold at the probe site was measured using a Harpenden caliper; participants with skinfolds > 15 mm were excluded (Perrey and Ferrari 2018).

### 2.12 Supplementation Protocol

Participants consumed 70 mL of nitrate‐rich BJ (∼6.4 mmol nitrate; Beet It Sport, James White Drinks Ltd., UK) or nitrate‐depleted BJ (< 0.05 mmol nitrate; Beet It Sport) at 07:00 (pre‐breakfast) and immediately after cognitive function in the second trial. This dose was selected based on evidence that an acute intake of ∼6–8 mmol nitrate can elevate plasma nitrite concentrations and enhance exercise performance (Demirli et al. 2025). CAF (3 mg/kg; Olimp Laboratories, Poland) or 250 mg sucrose was administered in an opaque capsule 60 minutes pre‐exercise (08:00) and immediately after cognitive function in the third trial. A 3 mg/kg CAF dose was chosen because it falls within the established ergogenic range (3–6 mg/kg), has been shown to enhance performance in judo and other combat sports (Delleli et al. 2022), and is associated with a lower incidence of adverse effects than higher doses (Goldstein et al. 2010). The familiarization session was conducted under a placebo.

### 2.13 Blood Sampling and Biochemical Analyses

Venous blood samples were collected at baseline (presupplementation), pre‐exercise (immediately before trial 1), mid‐exercise (after cognitive function in trial 3), prefinal SJFT (immediately before SJFT 6), and postexercise (5 minutes after the final SJFT). Whole blood pH was measured using a blood gas analyzer (Sysmex, Germany) immediately after collection at baseline and postexercise. Blood for plasma analyses was centrifuged at 2000 × g for 10 minutes at 4°C, and plasma aliquots were stored at −80°C for subsequent analysis; samples underwent no more than one freeze–thaw cycle. Plasma glucose was quantified using the enzymatic hexokinase method, in which hexokinase phosphorylates glucose and the resulting NADPH formation is measured spectrophotometrically. Blood lactate concentration was determined using a YSI 1500 Sport lactate analyzer (Yellow Springs Instruments, OH, USA) according to the manufacturer's instructions.

Plasma CAF concentration was determined by reversed‐phase high‐performance liquid chromatography with UV detection (HPLC‐UV). Plasma samples (200 μL) were mixed with 20 μL of internal standard solution (7‐*β*‐hydroxyethyl theophylline, 10 mg/L) and deproteinized by the addition of 400 μL acetonitrile. Samples were vortex‐mixed for 30 s and centrifuged at 14,000 × g for 10 minutes at 4°C. The supernatant was transferred to HPLC vials, and 20 μL was injected into the chromatographic system. Chromatographic separation was performed on a C18 reverse‐phase column (250 × 4.6 mm, 5 μm particle size) maintained at 30°C. The mobile phase consisted of water containing 0.1% acetic acid and acetonitrile (90:10, v/v) delivered at a flow rate of 1.0 mL/min with UV detection set at 273 nm. Under these conditions, the retention times were approximately 6.5 minutes for CAF and 8.1 minutes for the internal standard. Quantification was performed using external calibration curves prepared from certified CAF standards over the concentration range of 0.1–20.0 μg/mL (*r* ^2^ > 0.999). Quality‐control samples of low, medium, and high concentrations were analyzed with each batch. The assay demonstrated intra‐assay and inter‐assay coefficients of variation of < 5% and < 8%, respectively, with analytical recoveries exceeding 95%.

Nitrate and nitrite were assessed via an ozone‐based chemiluminescence assay (NOA‐280i, Sievers Instruments). For nitrate analysis, plasma samples were deproteinized by addition of ice‐cold ethanol, centrifuged at 14,000 g for 10 minutes, incubated for 30 minutes on ice, and centrifuged again; the deproteinized supernatant was reduced to NO with vanadium (III) chloride in concentrated HCl at 95°C and quantified against nitrate standards. For nitrite determination, non‐deproteinized plasma was reduced to NO using glacial acetic acid/potassium iodide and quantified by chemiluminescence. All assays included appropriate quality controls and standard curves.

### 2.14 Blinding Efficiency

Blinding efficacy was evaluated by a forced‐choice questionnaire in which participants identified which supplement they believed they had received after each session.

### 2.15 Statistical Analysis

Data are presented as mean ± standard deviation (SD). The Shapiro–Wilk test was used to assess the normality of the data distribution. Mauchly's test was also used to evaluate the sphericity assumption. If the sphericity assumption yielded a *p*‐value < 0.05, the Greenhouse–Geisser correction was applied. A two‐way repeated‐measures ANOVA (condition × time) was used to analyze performance, physiological, and cognitive variables. If the F‐ratio was significant, Bonferroni post hoc tests were applied. Effect size was reported as partial eta squared (*η*p^2^) with thresholds: < 0.01 trivial, 0.01–0.059 small, 0.06–0.139 moderate, and ≥ 0.14 large. Statistical significance was set at *p* < 0.05. Analyses were performed in SPSS Statistics (Version 27, IBM, USA).

## 3 Results

All participants completed the study and were included in the final analysis. No supplementation‐related side effects were reported during or after the experimental period.

Blinding efficacy was assessed after each experimental session by asking participants to identify the supplementation condition they believed they had received. The proportion of correct and incorrect guesses was as follows: BJ condition, 10 correct (28.6%) and 25 incorrect (71.4%); CAF condition, 9 correct (25.7%) and 26 incorrect (74.3%); BJCAF condition, 14 correct (40.0%) and 21 incorrect (60.0%); and PLA condition, 12 correct (34.3%) and 23 incorrect (65.7%).

A two‐way repeated‐measures ANOVA revealed a significant condition × time interaction for TT in the SJFT (*F*(15, 510) = 13.427, *p* < 0.001, *η*p^2^ = 0.28). Compared to PLA, TT was increased by BJ in SJFTs 5 (mean difference (MD) = 0.63, 95% CI [0.13, 1.13], *p* < 0.01) and 6 (MD = 0.86, 95% CI [0.35, 1.36], *p* < 0.001). TT was higher in the CAF condition than in PLA for SJFTs 1 (MD = 1.06, 95% CI [0.6, 1.52], *p* < 0.001), 2 (MD = 1.23, 95% CI [0.78, 1.79], *p* < 0.001), and 3 (MD = 1.03, 95% CI [0.5, 1.56], *p* < 0.001) and higher than BJ in SJFTs 1 (MD = 0.86, 95% CI [0.3, 1.43], *p* < 0.001) and 2 (MD = 0.97, 95% CI [0.3, 1.65], *p* < 0.01). In contrast, TT was lower in the CAF condition than in PLA for SJFT 5 (MD = −0.57, 95% CI [−1.14, −0.005], *p* < 0.05) and lower than both BJ and BJCAF in SJFTs 5 (BJ: MD = −1.2, 95% CI [−1.68, −0.71], *p* < 0.001; BJCAF: MD = −1.09, 95% CI [−1.64, −0.53], *p* < 0.001) and 6 (BJ: MD = −1.23, 95% CI [−1.85, −0.61], *p* < 0.001; BJCAF: MD = −0.83, 95% CI [−1.37, −0.28], *p* < 0.001). The BJCAF condition increased TT relative to PLA in SJFTs 1 (MD = 0.97, 95% CI [0.52, 1.42], *p* < 0.001), 2 (MD = 0.94, 95% CI [0.11, 1.77], *p* < 0.05), 3 (MD = 1, 95% CI [0.43, 1.57], *p* < 0.001), 5 (MD = 0.51, 95% CI [0.04, 0.99], *p* < 0.05), and 6 (MD = 0.46, 95% CI [0.12, 0.79], *p* < 0.01), and relative to BJ in SJFTs 1 (MD = 0.77, 95% CI [0.26, 1.29], *p* < 0.001) and 2 (MD = 0.63, 95% CI [0.06, 1.19], *p* < 0.05; Table 1).

**TABLE 1:** Effects of beetroot juice (BJ), caffeine (CAF), and beetroot juice + caffeine (BJCAF) supplementation on Special Judo Fitness Test (SJFT) outcomes.

		PLA	BJ	CAF	BJCAF
TT (*n*)	SJFT 1	27 ± 3	27 ± 3	28 ± 3^a^, ^b^	28 ± 3^a^, ^b^
	SJFT 2	26 ± 3	26 ± 3	27 ± 3^a^, ^b^	27 ± 3^a^, ^b^
	SJFT 3	25 ± 2	26 ± 3	26 ± 3^a^	26 ± 3^a^
	SJFT 4	25 ± 3	25 ± 3	25 ± 3	25 ± 3
	SJFT 5	24 ± 3	25 ± 3^a^, ^c^	23 ± 3^a^	25 ± 3^a^, ^c^
	SJFT 6	25 ± 3	25 ± 3^a^, ^c^	24 ± 3	25 ± 3^a^, ^c^
HR_0_ (beats/min)	SJFT 1	181 ± 3	180 ± 3	185 ± 4^a^, ^b^	184 ± 5^a^, ^b^
	SJFT 2	182 ± 3	181 ± 3	184 ± 5^a^, ^b^	184 ± 4^a^, ^b^
	SJFT 3	180 ± 4	180 ± 4	185 ± 4^a^, ^b^	184 ± 5^a^, ^b^
	SJFT 4	181 ± 4	181 ± 4	184 ± 4^a^, ^b^	185 ± 4^a^, ^b^
	SJFT 5	181 ± 3	181 ± 3	185 ± 4^a^, ^b^	184 ± 4^a^, ^b^
	SJFT 6	181 ± 3	181 ± 3	184 ± 3^a^, ^b^	184 ± 4^a^, ^b^
HR_1_ (beats/min)	SJFT 1	147 ± 7	144 ± 9	151 ± 6^b^, ^d^	148 ± 6
	SJFT 2	148 ± 7	145 ± 7^a^	151 ± 7^b^	149 ± 7^b^
	SJFT 3	147 ± 8	145 ± 5.8	152 ± 7^a^, ^b^, ^d^	148 ± 6^b^
	SJFT 4	148 ± 7	143 ± 7^a^	153 ± 7^a^, ^b^	151 ± 7^b^
	SJFT 5	148 ± 6	146 ± 8	154 ± 6^a^, ^b^	151 ± 7^b^
	SJFT 6	147 ± 8	141 ± 8^a^	151 ± 6^b^, ^d^	146 ± 8^b^
SJFT index	SJFT 1	12.44 ± 1.2	12.24 ± 1.4	12.25 ± 1.2	12.19 ± 1.3
	SJFT 2	12.81 ± 1.4	12.55 ± 1.6	12.39 ± 1.2^a^	12.55 ± 1.5
	SJFT 3	12.99 ± 1.3	12.65 ± 1.4^a^	12.91 ± 1.3	12.68 ± 1.3
	SJFT 4	13.4 ± 1.6	12.95 ± 1.5^a^, ^c^	13.6 ± 1.6	13.32 ± 1.4
	SJFT 5	13.88 ± 1.8	13.41 ± 1.7^a^, ^c^, ^d^	14.61 ± 1.8^a^	13.82 ± 1.7
	SJFT 6	13.5 ± 1.6	12.81 ± 1.6^a^, ^c^, ^d^	13.99 ± 1.7^a^	13.33 ± 1.5
RPE	SJFT 1	15 ± 1	15 ± 2	15 ± 2	15 ± 2
	SJFT 2	15 ± 2	16 ± 1	16 ± 2	15 ± 2
	SJFT 3	16 ± 1	16 ± 2	16 ± 2	16 ± 2
	SJFT 4	17 ± 1	17 ± 1	17 ± 2	17 ± 1
	SJFT 5	17 ± 2	17 ± 2	18 ± 1^a^, ^b^, ^d^	17 ± 2
	SJFT 6	15 ± 1	15 ± 2	16 ± 2^b^	16 ± 1

*Note:* Data are presented as mean ± standard deviation.

Abbreviations: HR_0_, heart rate immediately at the end of SJFT; HR_1_, heart rate 1 minute after SJFT; RPE, rate of perceived exertion; TT, total number of throws.

^a^ *p* < 0.05 significant difference from PLA.

^b^ *p* < 0.05 significant difference from BJ.

^c^ *p* < 0.05 significant difference from CAF.

^d^ *p* < 0.05 significant difference from BJCAF.

A condition × time interaction was observed for HR_0_ in the SJFT (*F* (5.80, 197.29) = 2.495, *p* < 0.05, *η*p^2^ = 0.07). HR_0_ was significantly higher in the CAF and BJCAF conditions than in the PLA (SJFT 1, CAF: MD = 4.2, 95% CI [2.39, 6.01], *p* < 0.001, and BJCAF: MD = 3.4, 95% CI [1.31, 5.5], *p* < 0.001; SJFT 2, CAF: MD = 1.89, 95% CI [0.08, 3.69], *p* < 0.05, and BJCAF: MD = 2.6, 95% CI [0.25, 4.95], *p* < 0.05; SJFT 3, CAF: MD = 4.51, 95% CI [2.6, 6.43], *p* < 0.001, and BJCAF: MD = 3.86, 95% CI [1.58, 6.14], *p* < 0.001; SJFT 4, CAF: MD = 3.46, 95% CI [1.62, 5.3], *p* < 0.001, and BJCAF: MD = 3.86, 95% CI [1.79, 5.92], *p* < 0.001; SJFT 5, CAF: MD = 3.74, 95% CI [2.19, 5.29], *p* < 0.001, and BJCAF: MD = 2.83, 95% CI [0.72, 4.93], *p* < 0.01; SJFT 6, CAF: MD = 3.11, 95% CI [1.65, 4.57], *p* < 0.001, and BJCAF: MD = 3.74, 95% CI [1.68, 5.81], *p* < 0.001) and BJ (SJFT 1, CAF: MD = 4.94, 95% CI [3.36, 6.52], *p* < 0.001, and BJCAF: MD = 4.14, 95% CI [2.12, 6.16], *p* < 0.001; SJFT 2, CAF: MD = 2.4, 95% CI [0.62, 4.18], *p* < 0.01, and BJCAF: MD = 3.11, 95% CI [1, 5.23], *p* < 0.001; SJFT 3, CAF: MD = 4.11, 95% CI [2.51, 5.72], *p* < 0.001, and BJCAF: MD = 3.46, 95% CI [1.15, 5.8], *p* < 0.001; SJFT 4, CAF: MD = 3.74, 95% CI [1.84, 5.65], *p* < 0.001, and BJCAF: MD = 4.14, 95% CI [1.98, 6.3], *p* < 0.001; SJFT 5, CAF: MD = 4.17, 95% CI [2.32, 6.02], *p* < 0.001, and BJCAF: MD = 3.26, 95% CI [1.47, 5.05], *p* < 0.01; SJFT 6, CAF: MD = 3.03, 95% CI [1.28, 4.77], *p* < 0.001, and BJCAF: MD = 3.66, 95% CI [1.68, 5.63], *p* < 0.001) conditions (Table 1).

For HR_1_ in the SJFT, a condition × time interaction was found (*F* (9.15, 311.12) = 2.259, *p* < 0.05, *η*p^2^ = 0.06). HR_1_ was higher in the CAF condition than in PLA for SJFTs 3 (MD = 4.57, 95% CI [0.31, 8.83], *p* < 0.05), 4 (MD = 4.6, 95% CI [0.43, 8.77], *p* < 0.05), and 5 (MD = 5.14, 95% CI [2.12, 8.17], *p* < 0.001), higher than BJ across all SJFTs (SJFT 1: MD = 6.66, 95% CI [2.19, 11.12], *p* < 0.001; SJFT 2: MD = 6.77, 95% CI [2.7, 10.85], *p* < 0.001; SJFT 3: MD = 7, 95% CI [3.79, 10.2], *p* < 0.001; SJFT 4: MD = 9.86, 95% CI [6.62, 13.1], *p* < 0.001; SJFT 5: MD = 7.77, 95% CI [4.02, 11.53], *p* < 0.001; SJFT 6: MD = 9.37, 95% CI [6.11, 12.63], *p* < 0.001), and higher than BJCAF in SJFTs 1 (MD = 2.83, 95% CI [1.08, 4.57], *p* < 0.001), 3 (MD = 3.71, 95% CI [1.31, 6.12], *p* < 0.001), and 6 (MD = 4.69, 95% CI [2.04, 7.33], *p* < 0.001). Furthermore, HR_1_ was lower in the BJ condition than in PLA for SJFTs 2 (MD = −3.09, 95% CI [−6.03, −0.14], *p* < 0.05), 4 (MD = −5.26, 95% CI [−8.37, −2.14], *p* < 0.001), and 6 (MD = −6.11, 95% CI [−10.4, −1.83], *p* < 0.01), and lower than BJCAF for SJFTs 2–6 (SJFT 2: MD = −4.51, 95% CI [−8.72, −0.31], *p* < 0.05; SJFT 3: MD = −3.29, 95% CI [−6.54, −0.03], *p* < 0.05; SJFT 4: MD = −8.17, 95% CI [−11.32, −5.02], *p* < 0.001; SJFT 5: MD = −5.51, 95% CI [−9.03, −2], *p* < 0.001; SJFT 6: MD = −4.69, 95% CI [−8.69, −0.68], *p* < 0.05; Table 1).

A significant condition × time interaction was found for the SJFT index (*F*(15, 510) = 12.826, *p* < 0.001, *η*p^2^ = 0.27). Compared to PLA, CAF improved the SJFT index in SJFT 2 (MD = −0.42, 95% CI [−0.71, −0.12], *p* < 0.01) but impaired it in SJFTs 5 (MD = 0.74, 95% CI [0.33, 1.15], *p* < 0.001) and 6 (MD = 0.49, 95% CI [0.23, 0.76], *p* < 0.001). BJ improved the index relative to PLA in SJFTs 3–6 (SJFT 3: MD = −0.33, 95% CI [−0.64, −0.02], *p* < 0.05; SJFT 4: MD = −0.45, 95% CI [−0.83, −0.08], *p* < 0.05; SJFT 5: MD = −0.47, 95% CI [−0.81, −0.12], *p* < 0.01; SJFT 6: MD = −0.68, 95% CI [−1.03, −0.33], *p* < 0.001), relative to CAF in SJFTs 4–6 (SJFT 4: MD = −0.65, 95% CI [−1.07, −0.23], *p* < 0.001; SJFT 5: MD = −1.21, 95% CI [−1.58, −0.83], *p* < 0.01; SJFT 6: MD = −1.18, 95% CI [−1.58, −0.77], *p* < 0.001), and relative to BJCAF in SJFTs 5 (MD = −0.41, 95% CI [−0.76, −0.06], *p* < 0.05) and 6 (MD = −0.52, 95% CI [−0.88, −0.15], *p* < 0.01). Additionally, the SJFT index was lower in the BJCAF condition than in CAF for SJFTs 5 (MD = −0.8, 95% CI [−1.19, −0.4], *p* < 0.001) and 6 (MD = −0.66, 95% CI [−1.02, −0.3], *p* < 0.001; Table 1).

A condition × time interaction was revealed for RPE (*F*(7.16, 243.22) = 2.516, *p* < 0.05, ηp^2^ = 0.07). RPE was significantly higher in the CAF condition than in PLA (MD = 0.49, 95% CI [0.35, 0.94], *p* < 0.05), BJ (MD = 0.69, 95% CI [0.24, 1.13], *p* < 0.001), and BJCAF (MD = 0.63, 95% CI [0.14, 1.12], *p* < 0.01) during SJFT 5 and higher than BJ in SJFT 6 (MD = 0.49, 95% CI [0.05, 0.92], *p* < 0.05; Table 1).

For CMJ height, a condition × time interaction was observed (*F*(15, 510) = 6.858, *p* < 0.001, *η*p^2^ = 0.17). Jump height decreased after all trials compared with pre‐exercise in every condition (*p* < 0.05). Relative to both PLA and BJ, CAF increased jump height at pre‐exercise (PLA: MD = 1.89, 95% CI [0.64, 3.13], *p* < 0.001; BJ: MD = 1.51, 95% CI [0.48, 2.54], *p* < 0.001) and in trials 1 (PLA: MD = 2.11, 95% CI [1.11, 3.11], *p* < 0.001; BJ: MD = 1.91, 95% CI [0.64, 3.18], *p* < 0.001), 2 (PLA: MD = 2.06, 95% CI [0.76, 3.36], *p* < 0.001; BJ: MD = 1.8, 95% CI [0.23, 3.37], *p* < 0.05), and 3 (PLA: MD = 2, 95% CI [1.24, 2.76], *p* < 0.001; BJ: MD = 1.2, 95% CI [0.33, 2.07], *p* < 0.01). BJCAF increased jump height at pre‐exercise (MD = 1.57, 95% CI [0.42, 2.73], *p* < 0.01) and in trials 1 to 5 (trial 1: MD = 2.23, 95% CI [1.04, 3.42], *p* < 0.001; trial 2: MD = 1.97, 95% CI [0.22, 3.72], *p* < 0.05; trial 3: MD = 2.4, 95% CI [1.15, 3.65], *p* < 0.001; trial 4: MD = 1.54, 95% CI [0.33, 2.75], *p* < 0.01; trial 5: MD = 1.51, 95% CI [0.45, 2.58], *p* < 0.01) compared to PLA, and at pre‐exercise (MD = 1.2, 95% CI [0.34, 2.06], *p* < 0.01) and in trials 1 to 3 (trial 1: MD = 2.03, 95% CI [1.05, 3], *p* < 0.001; trial 2: MD = 1.71, 95% CI [0.23, 3.2], *p* < 0.05; trial 3: MD = 1.6, 95% CI [0.48, 2.7], *p* < 0.01) compared to BJ. Jump height was also higher in the BJ condition than in PLA for trials 3 to 5 (trial 3: MD = 0.8, 95% CI [0.03, 1.57], *p* < 0.05; trial 4: MD = 1.06, 95% CI [0.12, 2], *p* < 0.05; trial 5: MD = 1, 95% CI [0.44, 1.56], *p* < 0.001). Conversely, CAF resulted in lower jump height than PLA (MD = −0.83, 95% CI [−1.47, −0.18], *p* < 0.01), BJ (MD = −1.83, 95% CI [−2.65, −1], *p* < 0.001) and BJCAF (MD = −2.34, 95% CI [−3.44, −1.25], *p* < 0.001) in trial 5 (Figure 3A).

**FIGURE 3:**
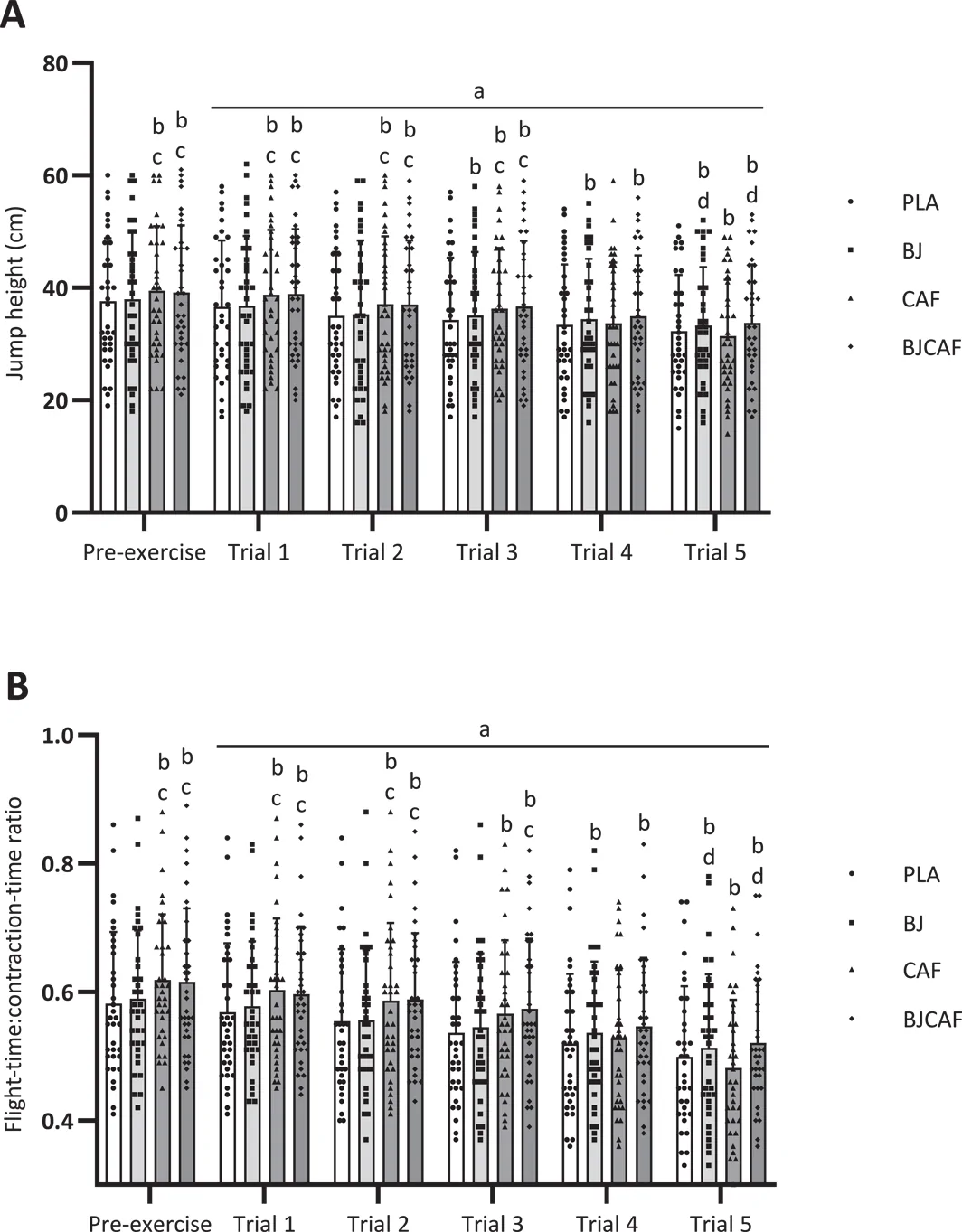
Effects of beetroot juice (BJ), caffeine (CAF), and beetroot juice + caffeine (BJCAF) supplementation on the jump height (A) and flight‐time/contraction‐time (FT:CT) ratio (B) in countermovement jump. ^a^ *p* < 0.05 significant difference from pre‐exercise; ^b^ *p* < 0.05 significant difference from PLA; ^c^ *p*< 0.05 significant difference from BJ; ^d^ *p* < 0.001 significant difference from CAF.

A condition × time interaction was also found for the FT:CT ratio in the CMJ (*F* (15, 510) = 10.658, *p* < 0.001, *η*p^2^ = 0.24). The FT:CT ratio decreased after all trials relative to pre‐exercise in all conditions (*p* < 0.05). CAF increased the ratio at pre‐exercise (MD = 0.04, 95% CI [0.02, 0.05], *p* < 0.01) and in trials 1 to 3 (trial 1: MD = 0.03, 95% CI [0.01, 0.05], *p* < 0.001; trial 2: MD = 0.03, 95% CI [0.006, 0.06], *p* < 0.01; trial 3: MD = 0.03, 95% CI [0.004, 0.06], *p* < 0.01) compared to PLA, and at pre‐exercise (MD = 0.03, 95% CI [0.01, 0.04], *p* < 0.001) and in trials 1 (MD = 0.02, 95% CI [0.001, 0.05], *p* < 0.01) and 2 (MD = 0.03, 95% CI [0.003, 0.06], *p* < 0.01) compared to BJ. BJCAF increased the ratio at pre‐exercise (MD = 0.03, 95% CI [0.01, 0.06], *p* < 0.01) and in trials 1 to 5 (trial 1: MD = 0.03, 95% CI [0.008, 0.05], *p* < 0.01; trial 2: MD = 0.03, 95% CI [0.01, 0.05], *p* < 0.001; trial 3: MD = 0.04, 95% CI [0.02, 0.06], *p* < 0.001; trial 4: MD = 0.03, 95% CI [0.01, 0.04], *p* < 0.001; trial 5: MD = 0.02, 95% CI [0.003, 0.04], *p* < 0.05) relative to PLA, and at pre‐exercise (MD = 0.03, 95% CI [0.001, 0.05], *p* < 0.05) and in trials 1 to 3 relative to BJ (trial 1: MD = 0.02, 95% CI [0.003, 0.04], *p* < 0.05; trial 2: MD = 0.03, 95% CI [0.01, 0.05], *p* < 0.001; trial 3: MD = 0.03, 95% CI [0.01, 0.05], *p* < 0.001). The ratio was also higher in the BJ condition than in PLA for trials 4 (MD = 0.02, 95% CI [0.001, 0.03], *p* < 0.05) and 5 (MD = 0.01, 95% CI [0.001, 0.03], *p* < 0.05). Furthermore, CAF decreased the FT:CT ratio in trial 5 compared to PLA (MD = −0.02, 95% CI [−0.03, −0.005], *p* < 0.01), BJ (MD = −0.03, 95% CI [−0.05, −0.02], *p* < 0.001) and BJCAF (MD = −0.04, 95% CI [−0.06, −0.02], *p* < 0.001; Figure 3B).

For HGS, a condition × time interaction was observed (*F*(15, 510) = 9.322, *p* < 0.001, ηp^2^ = 0.21). HGS decreased after all trials compared to pre‐exercise in every condition (*p* < 0.05). Relative to PLA and BJ, CAF increased HGS at pre‐exercise (PLA: MD = 2.23, 95% CI [1.24, 3.22], *p* < 0.001; BJ: MD = 1.77, 95% CI [0.93, 2.62], *p* < 0.001) and in trials 1 (PLA: MD = 1.49, 95% CI [0.69, 2.28], *p* < 0.001; BJ: MD = 1.54, 95% CI [0.52, 2.57], *p* < 0.001) and 2 (PLA: MD = 1.57, 95% CI [0.35, 2.8], *p* < 0.01; BJ: MD = 2.2, 95% CI [0.59, 3.81], *p* < 0.01). BJCAF increased HGS at pre‐exercise (MD = 2, 95% CI [1.08, 2.92], *p* < 0.001) and in trials 1 (MD = 1.17, 95% CI [0.38, 1.96], *p* < 0.001), 2 (MD = 1.17, 95% CI [0.11, 2.23], *p* < 0.05), and 4 (MD = 1.86, 95% CI [0.83, 2.88], *p* < 0.001) compared to PLA, and at pre‐exercise (MD = 1.54, 95% CI [0.6, 2.48], *p* < 0.001) and in trials 1 (MD = 1.23, 95% CI [0.33, 2.13], *p* < 0.01) and 2 (MD = 1.8, 95% CI [0.1, 3.49], *p* < 0.05) compared to BJ. HGS in the BJCAF condition was higher than in CAF for trials 4 (MD = 1.91, 95% CI [0.71, 3.12], *p* < 0.001) and 5 (MD = 2.06, 95% CI [0.6, 3.5], *p* < 0.01). In addition, HGS was higher in the BJ condition than in both PLA (MD = 1.14, 95% CI [0.13, 2.15], *p* < 0.05) and CAF (MD = 2.29, 95% CI [1, 3.57], *p* < 0.001) during trial 5 (Figure 4).

**FIGURE 4:**
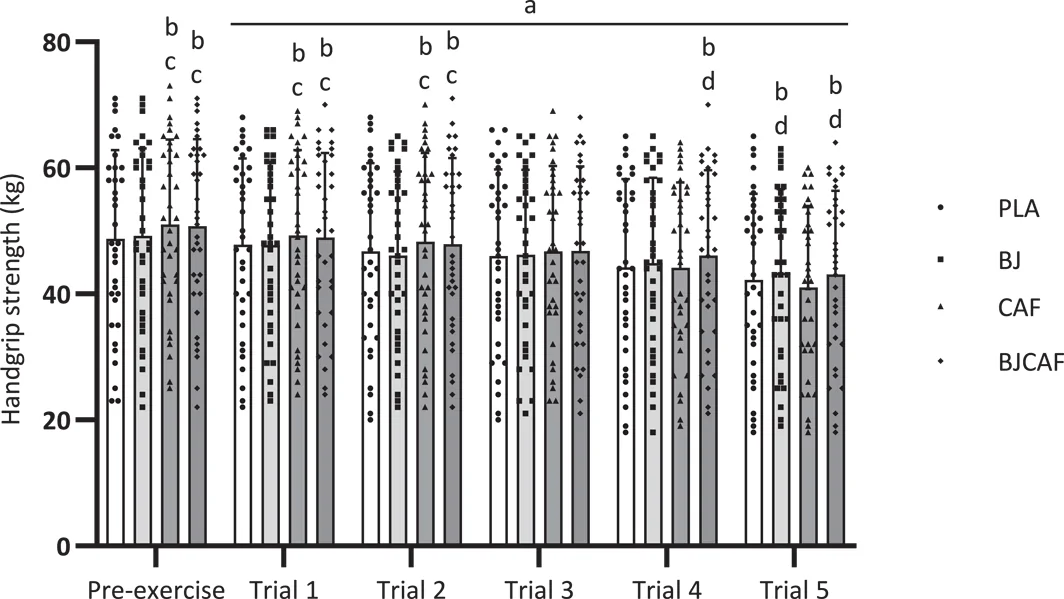
Effects of beetroot juice (BJ), caffeine (CAF), and beetroot juice + caffeine (BJCAF) supplementation on handgrip strength. ^a^ *p* < 0.05 significant difference from pre‐exercise; ^b^ *p* < 0.05 significant difference from PLA; ^c^ *p* < 0.05 significant difference from BJ; ^d^ *p* < 0.01 significant difference from CAF.

A condition × time interaction was found for the number of lapses in the PVT (*F*(15, 510) = 6.918, *p* < 0.001, ηp^2^ = 0.17). The number of lapses was lower in the CAF and BJCAF conditions than in PLA in pre‐exercise (CAF: MD = −0.6, 95% CI [−1.08, −0.12], *p* < 0.01; BJCAF: MD = −0.54, 95% CI [−1.05, −0.04], *p* < 0.05) and in trials 1 (CAF: MD = −1.57, 95% CI [−2.24, −0.9], *p* < 0.001; BJCAF: MD = −1.2, 95% CI [−1.97, −0.43], *p* < 0.001) and 2 (CAF: MD = −0.83, 95% CI [−1.5, −0.15], *p* < 0.01; BJCAF: MD = −91, 95% CI [−1.44, −0.38], *p* < 0.001). Similar results were also found in the comparison of CAF (pre‐exercise: MD = −0.69, 95% CI [−1.13, −0.24], *p* < 0.01; trial 1: MD = −1.49, 95% CI [−2.33, −0.64], *p* < 0.001; trial 2: MD = −089, 95% CI [−1.65, −0.13], *p* < 0.05) and BJCAF (pre‐exercise: MD = −0.63, 95% CI [−1.18, −0.08], *p* < 0.05; trial 1: MD = −1.11, 95% CI [−2.16, −0.07], *p* < 0.05; trial 2: MD = −0.97, 95% CI [−1.05, −0.89], *p* < 0.001) versus BJ. CAF increased the number of lapses compared to PLA in trial 5 (MD = 0.57, 95% CI [0.19, 0.96], *p* < 0.001) and compared to BJ and BJCAF in trials 4 (BJ: MD = 0.89, 95% CI [0.29, 1.48], *p* < 0.001; BJCAF: MD = 0.91, 95% CI [0.25, 1.58], *p* < 0.01) and 5 (BJ: MD = 1.09, 95% CI [0.35, 1.8], *p* < 0.001; BJCAF: MD = 1, 95% CI [0.33, 1.67], *p* < 0.001). BJ also resulted in lower lapses than PLA in trial 4 (MD = −0.43, 95% CI [−0.78, −0.08], *p* < 0.01; Figure 5A).

For mean response speed in the PVT, a condition × time interaction was observed (*F*(15, 510) = 8.122, *p* < 0.001, ηp^2^ = 0.19). CAF increased response speed compared to PLA at pre‐exercise (MD = 0.1, 95% CI [0.04, 0.15], *p* < 0.001) and in trials 1 (MD = 0.09, 95% CI [0.04, 0.15], *p* < 0.001), 2 (MD = 0.05, 95% CI [0.003, 0.1], *p* < 0.05), and 3 (MD = 0.05, 95% CI [0.009, 0.1], *p* < 0.05), and compared to BJ at pre‐exercise (MD = 0.1, 95% CI [0.07, 0.14], *p* < 0.001) and in trials 2 (MD = 0.06, 95% CI [0.007, 0.11], *p* < 0.05) and 3 (MD = 0.03, 95% CI [0.005, 0.05], *p* < 0.05). BJCAF increased mean response speed relative to PLA at pre‐exercise (MD = 0.07, 95% CI [0.01, 0.14], *p* < 0.05) and in trials 1 (MD = 0.08, 95% CI [0.02, 0.13], *p* < 0.01), 2 (MD = 0.04, 95% CI [0.006, 0.08], *p* < 0.05), and 4 (MD = 0.05, 95% CI [0.006, 0.09], *p* < 0.05), relative to BJ at pre‐exercise (MD = 0.08, 95% CI [0.04, 0.13], *p* < 0.001) and in trial 2 (MD = 0.05, 95% CI [0.001, 0.1], *p* < 0.05), and relative to CAF in trials 4 (MD = 0.06, 95% CI [0.01, 0.14], *p* < 0.05) and 5 (MD = 0.09, 95% CI [0.03, 0.15], *p* < 0.001). Furthermore, mean response speed was higher in the BJ condition than in PLA for trial 5 (MD = 0.07, 95% CI [0.04, 0.1], *p* < 0.001) and higher than CAF in trials 4 (MD = 0.06, 95% CI [0.003, 0.12], *p* < 0.05) and 5 (MD = 0.1, 95% CI [0.06, 0.16], *p* < 0.001). CAF decreased mean response speed compared to PLA in trial 5 (MD = −0.04, 95% CI [−0.07, −0.006], *p* < 0.05; Figure 5B).

**FIGURE 5:**
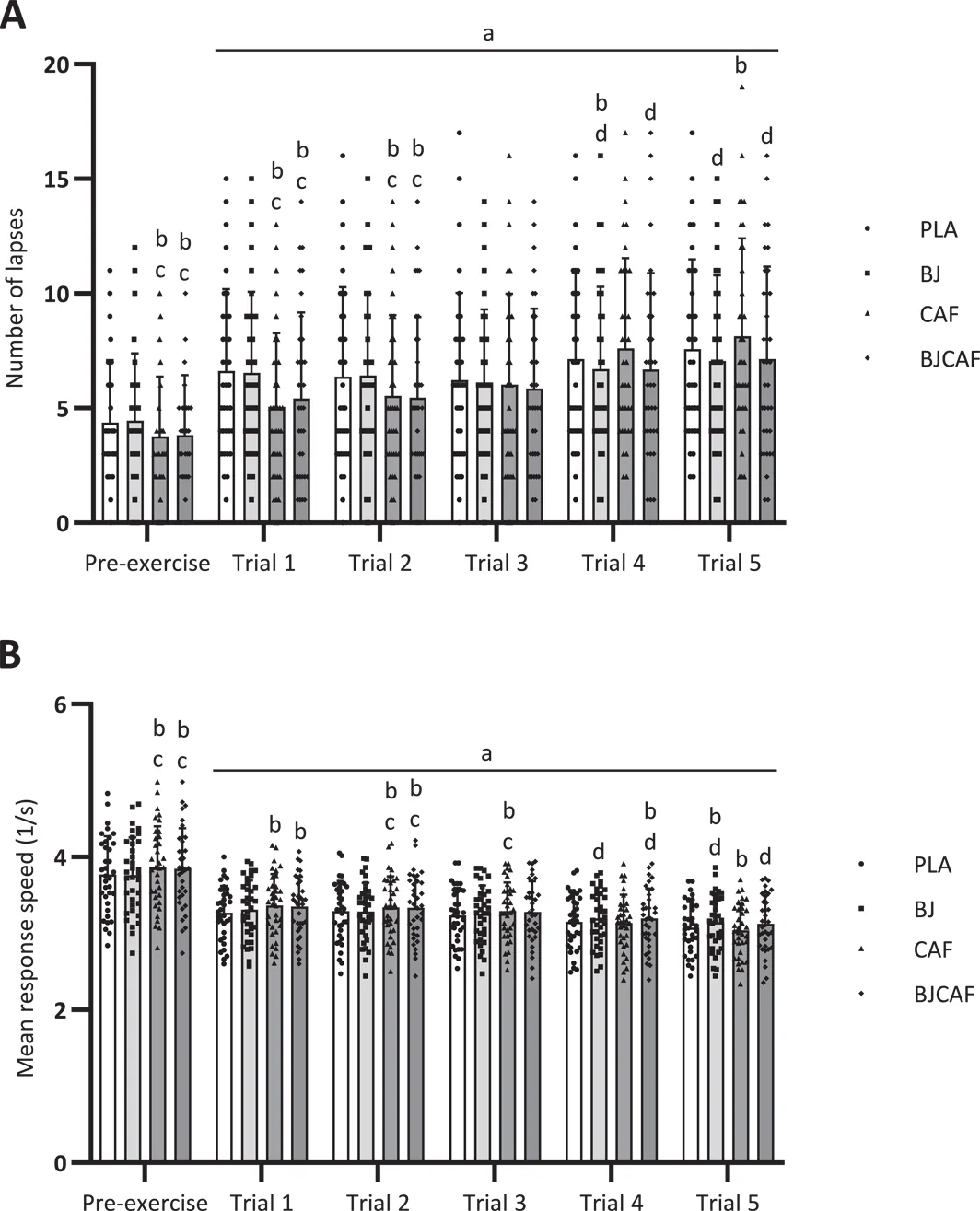
Effects of beetroot juice (BJ), caffeine (CAF), and beetroot juice + caffeine (BJCAF) supplementation on the cognitive performance outcomes. (A) Number of lapses. (B) Mean response speed. ^a^ *p* < 0.05 significant difference from pre‐exercise; ^b^ *p* < 0.05 significant difference from PLA; ^c^ *p* < 0.05 significant difference from BJ; ^d^ *p* < 0.05 significant difference from CAF.

A condition × time interaction was found for TSI during the final SJFT (*F*(6.15, 209.1) = 2.160, *p* < 0.05, ηp^2^ = 0.06). TSI decreased from baseline during the SJFT in all conditions (*p* < 0.001). Post‐test recovery TSI was higher in the BJ and BJCAF conditions compared to both PLA (BJ: MD = 2.5, 95% CI [0.96, 4.05], *p* < 0.001; BJCAF: MD = 1.91, 95% CI [1.22, 2.6], *p* < 0.001) and CAF (BJ: MD = 3.4, 95% CI [0.98, 45.81], *p* < 0.01; BJCAF: MD = 2.81, 95% CI [0.87, 4.74], *p* < 0.01; Figure 6).

**FIGURE 6:**
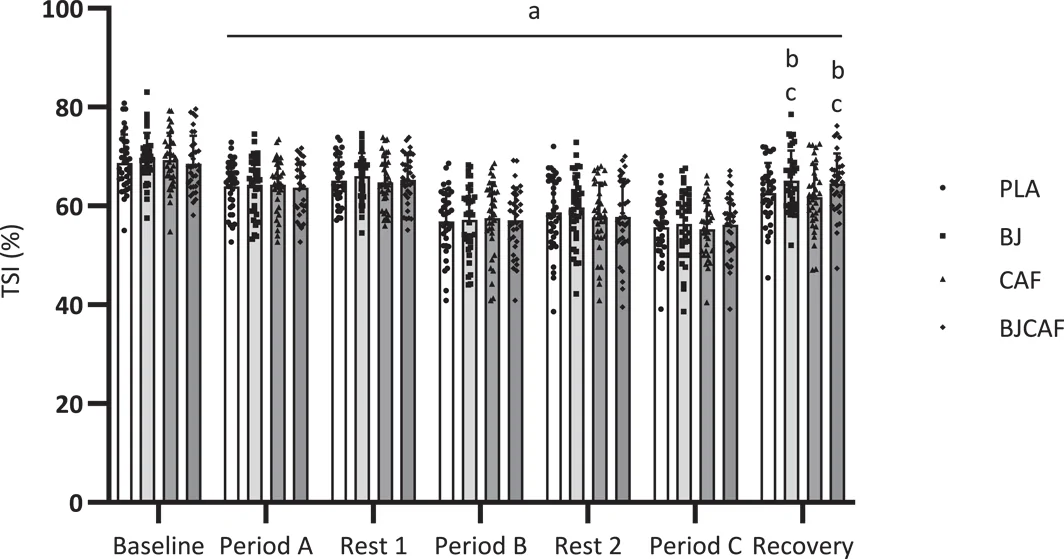
Effects of beetroot juice (BJ), caffeine (CAF), and beetroot juice + caffeine (BJCAF) supplementation on vastus lateralis oxygenation as measured by the tissue saturation index (TSI). ^a^ *p* < 0.001 significant difference from baseline; ^b^ *p* < 0.001 significant difference from PLA; ^c^ *p* < 0.01 significant difference from CAF.

A condition × time interaction was observed for plasma lactate (*F*(12, 408) = 9.693, *p* < 0.001, ηp^2^ = 0.22). Lactate concentration was elevated at mid‐exercise, prefinal SJFT, and postexercise relative to baseline in all conditions (*p* < 0.001). Lactate was higher in the CAF than in PLA and BJ conditions at mid‐exercise (PLA: MD = 1, 95% CI [0.51, 1.49], *p* < 0.001; BJ: MD = 1.17, 95% CI [0.63, 1.7], *p* < 0.001) and postexercise (PLA: MD = 1.24, 95% CI [0.54, 1.94], *p* < 0.001; BJ: MD = 0.95, 95% CI [0.21, 1.7], *p* < 0.01). Also, BJCAF led to elevated lactate relative to PLA and BJ at mid‐exercise (PLA: MD = 0.72, 95% CI [0.13, 1.31], *p* < 0.01; BJ: MD = 0.88, 95% CI [0.38, 1.38], *p* < 0.001) and postexercise (PLA: MD = 1.02, 95% CI [0.52, 1.51], *p* < 0.001; BJ: MD = 0.73, 95% CI [0.1, 1.36], *p* < 0.05). At prefinal SJFT, lactate was higher in CAF than in PLA (MD = 1.12, 95% CI [0.78, 1.46], *p* < 0.001), BJ (MD = 1.53, 95% CI [1.01, 2.04], *p* < 0.001), and BJCAF (MD = 0.98, 95% CI [0.43, 1.52], *p* < 0.001), while BJ resulted in lower lactate than both PLA (MD = −0.41, 95% CI [−0.77, −0.04], *p* < 0.05) and BJCAF (MD = −0.55, 95% CI [−0.92, −0.17], *p* < 0.01; Table 2).

**TABLE 2:** Effects of beetroot juice (BJ), caffeine (CAF), and beetroot juice + caffeine (BJCAF) supplementation on blood variables.

		PLA	BJ	CAF	BJCAF
Lactate (mmol/L)	Baseline	1.19 ± 0.3	1.32 ± 0.37	1.28 ± 0.3	1.22 ± 0.31
	Pre‐exercise	1.25 ± 0.32	1.34 ± 0.32	1.36 ± 0.36	1.27 ± 0.28
	Mid‐exercise	13.33 ± 1.5^a^	13.17 ± 2^a^	14.33 ± 1.5^a^, ^b^, ^c^	14.05 ± 1.7^a^, ^b^, ^c^
	Pre‐final SJFT	3.81 ± 1^a^	3.4 ± 1^a^, ^b^	4.93 ± 1.3^a^, ^b^, ^c^, ^e^	3.95 ± 1.1^a^, ^c^
	Post‐exercise	10.38 ± 2.1^a^	10.67 ± 2.1^a^	11.62 ± 2.3^a^, ^b^, ^c^	11.39 ± 1.7^a^, ^b^, ^c^
Glucose (mmol/L)	Baseline	4.47 ± 0.36	4.39 ± 0.39	4.45 ± 0.41	4.4 ± 0.43
	Pre‐exercise	5.13 ± 0.57^a^	5.17 ± 0.6^a^	5.37 ± 0.58^a^	5.21 ± 0.54^a^, ^c^
	Mid‐exercise	4.86 ± 0.5^a^	4.73 ± 0.57^a^	5.31 ± 0.57^a^, ^b^, ^c^	5.09 ± 0.55^a^, ^c^
	Pre‐final SJFT	4.73 ± 0.51^a^	4.59 ± 0.58	5.21 ± 0.63^a^, ^b^, ^c^, ^e^	4.92 ± 0.53^a^, ^b^, ^c^
	Post‐exercise	4.84 ± 0.52^a^	4.68 ± 0.6^a^	5.32 ± 0.71^a^, ^b^, ^c^	5.18 ± 0.54^a^, ^c^
Nitrate (μM)	Baseline	35.66 ± 9.7	36.26 ± 10.6	37.57 ± 8.6	37.03 ± 11.5
	Pre‐exercise	36.03 ± 9.8	437.37 ± 171^a^, ^b^, ^d^	38.54 ± 10.4	443.66 ± 185^a^, ^b^, ^d^
	Mid‐exercise	40.29 ± 13.1	446.37 ± 184^a^, ^b^, ^d^	41.6 ± 9.4	439.34 ± 176^a^, ^b^, ^d^
	Pre‐final SJFT	37.14 ± 11.7	452.06 ± 182^a^, ^b^, ^d^	37.69 ± 8.8	449.09 ± 177^a^, ^b^, ^d^
	Post‐exercise	38.8 ± 13.8	444.63 ± 183^a^, ^b^, ^d^	38.14 ± 9.2	446.09 ± 186^a^, ^b^, ^d^
Nitrite (nM)	Baseline	65.66 ± 13.6	61 ± 12.8	58.86 ± 15.6	63.23 ± 15.7
	Pre‐exercise	61.83 ± 11.9	164.83 ± 40.7^a^, ^b^, ^d^	62.63 ± 16.1	152.17 ± 36.8^a^, ^b^, ^d^
	Mid‐exercise	69.97 ± 14.4	160.14 ± 35^a^, ^b^, ^d^	65.09 ± 17.8	154.51 ± 39^a^, ^b^, ^d^
	Pre‐final SJFT	67.4 ± 10.5	153.57 ± 33.4^a^, ^b^, ^d^	69.49 ± 17.5	159.49 ± 42.8^a^, ^b^, ^d^
	Post‐exercise	59.74 ± 12.8	160.29 ± 35.2^a^, ^b^, ^d^	64.54 ± 19.9	156.69 ± 40.7^a^, ^b^, ^d^
Caffeine (μg/ml)	Baseline	BQL	BQL	BQL	BQL
	Pre‐exercise	BQL	BQL	3.11 ± 0.98^a^, ^b^, ^d^	3.33 ± 1.1^a^, ^b^, ^d^
	Mid‐exercise	BQL	BQL	3.37 ± 1^a^, ^b^, ^d^	3.26 ± 1^a^, ^b^, ^d^
	Pre‐final SJFT	BQL	BQL	3.62 ± 1.1^a^, ^b^, ^d^	3.49 ± 1^a^, ^b^, ^d^
	Post‐exercise	BQL	BQL	3.71 ± 0.97^a^, ^b^, ^d^	3.65 ± 1.1^a^, ^b^, ^d^
pH	Baseline	7.39 ± 0.03	7.41 ± 0.03	7.4 ± 0.03	7.41 ± 0.02
	Pre‐exercise	—	—	—	—
	Mid‐exercise	—	—	—	—
	Pre‐final SJFT	—	—	—	—
	Post‐exercise	7.2 ± 0.05^a^	7.25 ± 0.07^a^, ^b^, ^d^, ^e^	7.15 ± 0.06^a^, ^b^, ^e^	7.18 ± 0.05^a^

*Note:* Data are presented as mean ± standard deviation.

Abbreviation: BQL, below quantification levels.

^a^ *p* < 0.05 significant difference from baseline.

^b^ *p* < 0.05 significant difference from PLA.

^c^ *p* < 0.05 significant difference from BJ.

^d^ *p* < 0.05 significant difference from CAF.

^e^ *p* < 0.05 significant difference from BJCAF.

For plasma glucose, a condition × time interaction was found (*F*(4.7, 159.1) = 6.815, *p* < 0.05, ηp^2^ = 0.17). Glucose levels were elevated at preexercise, mid‐exercise, prefinal SJFT, and postexercise compared to baseline in all conditions (*p* < 0.05), except in the BJ condition at prefinal SJFT (*p* = 0.092). Glucose was significantly higher in the CAF condition than in PLA and BJ at mid‐exercise (PLA: MD = 0.44, 95% CI [0.09, 0.79], *p* < 0.01; BJ: MD = 0.57, 95% CI [0.28, 0.87], *p* < 0.001), prefinal SJFT (PLA: MD = 0.48, 95% CI [0.11, 0.84], *p* < 0.01; BJ: MD = 0.62, 95% CI [0.32, 0.92], *p* < 0.001), and postexercise (PLA: MD = 0.48, 95% CI [0.06, 0.9], *p* < 0.05; BJ: MD = 0.64, 95% CI [0.29, 0.99], *p* < 0.001), and higher than BJCAF at prefinal SJFT (MD = 0.29, 95% CI [0.04, 0.54], *p* < 0.05). BJCAF also resulted in higher glucose levels than BJ at mid‐exercise (MD = 0.35, 95% CI [0.04, 0.67], *p* < 0.05), prefinal SJFT (MD = 0.33, 95% CI [0.01, 0.65], *p* < 0.05), and postexercise (MD = 0.5, 95% CI [0.21, 0.79], *p* < 0.001; Table 2).

Significant condition × time interactions were found for plasma nitrate (*F*(12, 408) = 147.612, *p* < 0.001, ηp^2^ = 0.81) and nitrite (*F*(4.05, 137.58) = 173.319, *p* < 0.001, ηp^2^ = 0.84). BJ and BJCAF increased plasma nitrate and nitrite concentrations at pre‐exercise, mid‐exercise, prefinal SJFT, and postexercise relative to baseline (*p* < 0.001). Nitrate and nitrite levels were higher in the BJ (nitrate: pre‐exercise, MD = 401.34, 95% CI [318.49, 484.19], *p* < 0.001; mid‐exercise, MD = 406.09, 95% CI [318.69, 493.48], *p* < 0.001; prefinal SJFT, MD = 414.914, 95% CI [328.61, 501.22], *p* < 0.001; postexercise, MD = 405.83, 95% CI [320.43, 491.23], *p* < 0.001; nitrite: pre‐exercise, MD = 103, 95% CI [87.27, 118.73], *p* < 0.001; mid‐exercise, MD = 90.17, 95% CI [77.99, 102.35], *p* < 0.001; prefinal SJFT, MD = 86.17, 95% CI [73.29, 99.05], *p* < 0.001; postexercise, MD = 100.54, 95% CI [87.83, 113.25], *p* < 0.001) and BJCAF (nitrate: pre‐exercise, MD = 407.63, 95% CI [317.84, 497.42], *p* < 0.001; mid‐exercise, MD = 399.06, 95% CI [315.16, 482.95], *p* < 0.001; prefinal SJFT, MD = 411.94, 95% CI [328.03, 495.86], *p* < 0.001; postexercise, MD = 407.29, 95% CI [319.78, 494.79], *p* < 0.001; nitrite: pre‐exercise, MD = 90.34, 95% CI [76.36, 104.33], *p* < 0.001; mid‐exercise, MD = 84.54, 95% CI [71, 98.09], *p* < 0.001; prefinal SJFT, MD = 92.09, 95% CI [74.34, 109.84], *p* < 0.001; postexercise, MD = 96.94, 95% CI [81.98, 111.9], *p* < 0.001) conditions than in PLA. Similar results were also observed comparing BJ (nitrate: pre‐exercise, MD = 398.83, 95% CI [317.11, 480.54], *p* < 0.001; mid‐exercise, MD = 404.77, 95% CI [317.13, 492.41], *p* < 0.001; prefinal SJFT, MD = 414.371, 95% CI [327.07, 501.68], *p* < 0.001; postexercise, MD = 406.49, 95% CI [318.84, 494.13], *p* < 0.001; nitrite: pre‐exercise, MD = 102.2, 95% CI [87.31, 117.09], *p* < 0.001; mid‐exercise, MD = 95.06, 95% CI [82.68, 107.44], *p* < 0.001; prefinal SJFT, MD = 84.09, 95% CI [72.58, 95.59], *p* < 0.001; postexercise, MD = 95.74, 95% CI [84.63, 106.86], *p* < 0.001) and BJCAF (nitrate: pre‐exercise, MD = 405.11, 95% CI [317, 493.23], *p* < 0.001; mid‐exercise, MD = 397.74, 95% CI [313.93, 481.55], *p* < 0.001; prefinal SJFT, MD = 411.4, 95% CI [326.67, 496.13], *p* < 0.001; postexercise, MD = 407.94, 95% CI [318.21, 497.68], *p* < 0.001; nitrite: pre‐exercise, MD = 89.54, 95% CI [77.52, 101.56], *p* < 0.001; mid‐exercise, MD = 89.43, 95% CI [75.74, 103.12], *p* < 0.001; prefinal SJFT, MD = 90, 95% CI [75.66, 104.34], *p* < 0.001; postexercise, MD = 92.14, 95% CI [79.51, 104.77], *p* < 0.001) with the CAF condition (Table 2).

A condition × time interaction was observed for plasma CAF (*F*(12, 408) = 235.976, *p* < 0.001, ηp^2^ = 0.87). Plasma CAF concentration increased from baseline at pre‐exercise, mid‐exercise, prefinal SJFT, and post‐exercise in the CAF and BJCAF conditions (*p* < 0.001). Levels were higher in the CAF than in PLA (pre‐exercise: MD = 3.11, 95% CI [2.64, 3.57], *p* < 0.001; mid‐exercise: MD = 3.37, 95% CI [2.87, 3.87], *p* < 0.001; prefinal SJFT: MD = 3.62, 95% CI [3.1, 4.16], *p* < 0.001; postexercise: MD = 3.71, 95% CI [3.25, 4.17], *p* < 0.001) and BJ (pre‐exercise: MD = 3.1, 95% CI [2.64, 3.57], *p* < 0.001; mid‐exercise: MD = 3.37, 95% CI [2.87, 3.86], *p* < 0.001; prefinal SJFT: MD = 3.6, 95% CI [3.08, 4.15], *p* < 0.001; postexercise: MD = 3.7, 95% CI [3.21, 4.05], *p* < 0.001). The BJCAF also led to a higher plasma CAF concentration than PLA (pre‐exercise: MD = 3.33, 95% CI [2.78, 3.87], *p* < 0.001; mid‐exercise: MD = 3.26, 95% CI [2.78, 3.73], *p* < 0.001; prefinal SJFT: MD = 3.48, 95% CI [3.1, 3.96], *p* < 0.001; postexercise: MD = 3.65, 95% CI [3.11, 4.18], *p* < 0.001) and BJ (pre‐exercise: MD = 3.22, 95% CI [2.82, 3.81], *p* < 0.001; mid‐exercise: MD = 3.14, 95% CI [2.62, 3.70], *p* < 0.001; prefinal SJFT: MD = 3.42, 95% CI [3, 3.84], *p* < 0.001; postexercise: MD = 3.53, 95% CI [3.18, 4.17], *p* < 0.001; Table 2).

A condition × time interaction for whole‐blood pH was observed (*F*(3, 102) = 32.125, *p* > 0.001, ηp^2^ = 0.49). There were no significant differences between conditions at baseline (*p* > 0.05). Whole‐blood pH decreased from baseline to posttest in all conditions (*p* > 0.001). Post‐exercise whole‐blood pH was higher in the BJ condition than in PLA (MD = 0.05, 95% CI [0.03, 0.07], *p* < 0.001), CAF (MD = 0.1, 95% CI [0.07, 0.12], *p* < 0.001), and BJCAF (MD = 0.07, 95% CI [0.04, 0.09], *p* < 0.001). In addition, CAF produced lower post‐exercise pH than PLA (MD = −0.04, 95% CI [−0.06, −0.03], *p* < 0.001) and BJCAF (MD = −0.03, 95% CI [−0.05, −0.01], *p* < 0.01; Table 2).

## 4 Discussion

This investigation provides novel insights into the dynamic and temporal effects of acute CAF and BR supplementation on sport‐specific performance, physiological responses, and recovery across a simulated judo competition day. Our findings demonstrate that while acute 3 mg/kg CAF enhances early‐trial performance, its benefits decline progressively across repeated high‐intensity efforts. In contrast, BJ supplementation exhibited sustained efficacy in later trials. Importantly, the combined supplementation effectively preserved performance during the later stages of the simulated competition day while maintaining the early‐trial benefits observed with CAF. These results confirm our initial hypotheses and underscore the necessity of considering accumulated tournament fatigue when evaluating nutritional ergogenic aids, representing a critical shift from single‐bout paradigms toward more ecologically valid models.

To facilitate visualization of within‐participant trajectories across the four supplementation conditions, Supporting Information S1: Figure S1 presents paired individual data with connecting lines. Our results revealed an apparent biphasic performance pattern in the CAF condition, characterized by improved SJFT performance during SJFTs 1–3, whereas this ergogenic effect was not maintained during SJFT 5. Although numerous studies have reported the ergogenic effects of CAF during single bouts of judo‐specific (Durkalec‐Michalski et al. 2019) and other high‐intensity exercise (Ouergui et al. 2023), fewer studies have examined its efficacy across repeated bouts performed over an extended period. Our observation aligns with evidence suggesting that the ergogenic effects of CAF may attenuate during the later stages of prolonged exercise (Crowe et al. 2006; Greer et al. 1998). However, it should also be acknowledged that our findings contrast with a substantial body of evidence showing that CAF can enhance repeated high‐intensity exercise performance and attenuate fatigue across multiple exercise bouts, including repeated‐sprint and interval‐exercise protocols (Carr et al. 2008; Belbis et al. 2022; Feng et al. 2025). Therefore, the later‐session decline observed in the CAF condition is unlikely to reflect a generalized ergolytic effect of CAF. Rather, it may be specific to the prolonged, judo‐specific nature of our protocol, which combined repeated high‐intensity randori bouts, sport‐specific fitness testing, and cognitive tasks over a ∼3.5‐h simulated competition day. For example, Feng et al. (2025) reported an anti‐fatigue effect of CAF following a standardized 50‐min fatigue protocol in elite badminton players. However, their protocol produced no significant differences in HR or RPE between conditions, whereas our protocol elicited substantially greater cumulative metabolic and perceptual stress. Collectively, these findings suggest that the decline observed in the later stages of the CAF condition may reflect the unique physiological and perceptual demands of prolonged competition‐like stress rather than a universal negative effect of CAF during repeated exercise.

An important consideration when interpreting the present findings is that the 4‐min randori and the SJFT, although both designed to reproduce key high‐intensity demands of judo, impose different relative metabolic demands. Energy‐system analyses indicate that simulated judo matches rely predominantly on oxidative metabolism when the entire bout is considered, with Julio et al. (2017) estimating contributions of approximately 70% oxidative, 21% ATP‐PCr, and 8% glycolytic metabolism. In contrast, the SJFT places a greater relative demand on anaerobic energy provision because of its repeated maximal throwing efforts and very short recovery intervals; Franchini et al. (2011) reported contributions of approximately 42% ATP‐PCr, 30% glycolytic, and 28% aerobic metabolism during the SJFT. Thus, the present protocol should not be viewed as repeatedly reproducing the metabolic profile of a judo match per se. Rather, the sequential combination of 4‐min randori and SJFT imposed a combination of substantial oxidative stress from the randori and repeated high‐rate anaerobic energy demands from the SJFT. This distinction may be particularly relevant to the progressive fatigue observed across the simulated competition day and to the differential responses to CAF and BJ, as the ability to repeatedly restore phosphocreatine, manage glycolytic‐derived metabolic perturbations, and maintain oxidative energy production would become increasingly important across successive trials.

The present findings should also be interpreted in the context of the CAF dose employed. Although higher CAF doses have been reported to enhance judo‐specific performance (Durkalec‐Michalski et al. 2019), our participants were low habitual CAF consumers and underwent a prolonged CAF‐restriction period before testing. This factor may partly explain differences between the present findings and studies involving habitual or moderate‐to‐high CAF consumers, whose physiological and performance responses to supplementation may differ. It is likely that this restriction period increased sensitivity to CAF supplementation (Khodadadi et al. 2025) and contributed to the ergogenic response observed during the initial trials. However, given the already low habitual CAF intake of our participants (≤ 0.99 mg/kg/day), the magnitude of any withdrawal‐ or resensitization‐related effect is difficult to determine. Furthermore, the 3 mg/kg dose improved SJFT performance during the initial trials, demonstrating that it was sufficient to produce ergogenic effects in this cohort. Therefore, the later‐session decline observed in the CAF condition should not be interpreted as evidence that low‐dose CAF is ineffective; rather, it may reflect an interaction between CAF dose, participant characteristics, and the prolonged demands of the simulated competition protocol. Although higher CAF doses may elicit different responses during prolonged competition day, the present findings cannot be attributed to declining CAF availability because CAF was administered both before and during the protocol, and plasma CAF concentrations remained elevated throughout the simulated competition day.

The mechanisms underlying the attenuation of the ergogenic effect of CAF during the later stages of the protocol are likely multifactorial. A primary molecular explanation involves the accumulation of extracellular adenosine during repeated intense exercise. Although CAF competitively blocks adenosine receptors (primarily A_1_ and A_2A_), high‐intensity exercise accelerates ATP breakdown, elevating extracellular adenosine levels (Langberg et al. 2002). As the CAF‐to‐adenosine ratio decreases, CAF's receptor occupancy falls. The elevated adenosine concentration then outcompetes CAF for binding, which may precipitate a sharp increase in perceived fatigue and reduced central motor drive. This explanation is largely supported by our PVT outcomes. During preexercise and initial trials, CAF reduced lapses and increased response speed, reflecting its well‐known psychoactive benefits (Ruxton 2008). However, the significant impairment in CAF's cognitive performance in trial 5 indicates the development of central fatigue. This rebound effect is particularly problematic in judo tournaments, where repeated 4‐minute randori bouts impose substantial metabolic and neural loads (Campos et al. 2025; Detanico et al. 2015; Stavrinou et al. 2016). In a critical competition, such cognitive decline could lead to missed opportunities or tactical mistakes, resulting in defeat. Concurrently, the significantly higher plasma lactate and lower postexercise pH in the CAF condition indicate a greater reliance on anaerobic glycolysis and consequent metabolic acidosis, which can contribute to muscle fatigue and impaired technical execution (Franchini et al. 2013; James et al. 2016). While CAF is known to enhance alertness and may increase neural drive and motor unit recruitment, thereby improving force production and exercise performance, the direct stimulation of sarcoplasmic reticulum calcium release observed in vitro (Davis and Green 2009) is unlikely to occur to a meaningful extent at the plasma CAF concentrations typically achieved following supplementation in humans. Nevertheless, the enhanced exercise intensity facilitated by CAF may increase glycolytic flux and contribute to greater lactate accumulation and hydrogen ion production during repeated high‐intensity efforts (Davis and Green 2009; Laurent et al. 2000). The exacerbated metabolic disturbance, coupled with a significantly higher RPE in later trials, likely overshadowed any persisting central stimulatory benefits, leading to the observed performance drop. This explanation is further supported by the decline in CMJ height and FT:CT ratio in the CAF condition at trial 5, indicating profound neuromuscular fatigue.

Unlike CAF, BJ did not significantly increase TT in the early SJFTs but conferred a clear advantage in later SJFTs (SJFTs 5 and 6). The late improvement in the TT and SJFT index with BJ, together with the preservation of neuromuscular function (jump height, FT:CT ratio, and HGS) in the later trials, points to reduced peripheral fatigue, likely mediated by enhanced skeletal muscle calcium handling, mitochondrial efficiency, and oxygen delivery (Clements et al. 2014; Larsen et al. 2007; Vanhatalo et al. 2011). These results agree with studies showing that dietary nitrate attenuates the decline in repeated‐sprint performance and improves recovery (Thompson et al. 2015), and extend that evidence to the complex, intermittent demands of judo competition.

The decrement in HGS observed in our study is consistent with the well‐documented fatigue profile of judo competition. Successive judo bouts have been shown to reduce maximal isometric HGS in both hands, with the dominant hand showing significant decreases across multiple bouts, accompanied by progressive increases in perceived exertion (Bonitch‐Domínguez et al. 2010). This pattern aligns with our observation of reduced HGS in the later trials, particularly under CAF supplementation. The inverse relationship between postbout HGS and blood lactate concentration reported in the literature (Bonitch‐Domínguez et al. 2010) further supports our interpretation that the greater metabolic disturbance in the CAF condition likely contributed to the larger HGS decrement, whereas preserved HGS in the BJ and BJCAF conditions may reflect reduced peripheral fatigue.

To contextualize the magnitude of the observed performance changes, our SJFT 1 outcomes are comparable to previously reported values in senior male and female judo athletes. According to the normative classification tables proposed by Sterkowicz‐Przybycień et al. (2019, 2014), our SJFT 1 values fall within the expected range for competitive‐trained judo athletes, supporting the ecological validity of our simulated competition protocol.

Improved cardiovascular efficiency and faster reoxygenation between bouts are indicated by the lower HR1 values across SJFTs and the faster posttest TSI recovery observed following BJ. These findings are consistent with improved microvascular function and improved matching of oxygen delivery to utilization (Hirai et al. 2012). These adaptations are likely mechanistically related to NO‐mediated vasodilation, which preferentially increases blood flow to active type II fibers recruited during high‐intensity intermittent exercise (Ferguson et al. 2013). Faster oxidative ATP synthesis and phosphocreatine resynthesis support subsequent performance. Furthermore, NO can reduce the energetic cost of force production and enhance excitation–contraction coupling (Fulford et al. 2013; Stamler and Meissner 2001), which could explain why performance is maintained even in reduced cardiac responses.

The blood lactate responses observed in our study are consistent with the characteristic metabolic profile of judo competition. Peak blood lactate concentrations in judo matches often reach ∼12–15 mmol/L (Bonitch‐Domínguez et al. 2010; Degoutte et al. 2003), and clearance is relatively slow, with values remaining clearly elevated 15 minutes after competition (Bonitch‐Domínguez et al. 2010; Franchini et al. 2003). Therefore, the time course of lactate clearance observed in our simulation, with elevated levels persisting into recovery, is in line with both simulated and official judo matches.

The lower blood lactate at the prefinal SJFT and the higher whole‐blood pH postexercise in the BJ condition suggest reduced reliance on anaerobic glycolysis and better systemic acid–base homeostasis. Enhanced mitochondrial function and oxidative metabolism would preserve glycolytic capacity and limit H^+^ accumulation, consistent with a prior mechanistic study (Vaughan et al. 2016). The absence of early‐trial advantages with BJ implies that its ergogenic effects may require accumulating metabolic stress to become evident, which aligns with the preferential activation of the nitrate‐nitrite‐NO pathway under hypoxic and acidotic conditions (Jones 2014; Kim‐Shapiro and Gladwin 2014).

Interestingly, the combined BJCAF condition provided the early‐trial benefits typical of CAF while preserving the late‐trial advantages associated with BJ, resulting in the best overall SJFT performance (Supporting Information S1: Figure S2) and the most favorable neuromuscular outcomes (Supporting Information S1: Figures S3–S5). This pattern suggests complementary rather than simply additive effects. Specifically, the combination increased TT across almost all SJFTs versus placebo and attenuated the late‐session performance decrement observed with CAF alone in SJFTs 5 and 6. Current evidence on combined CAF and nitrate ingestion is mixed. For example, Lane et al. (2014) reported no additional benefit of co‐ingestion of BJ and CAF in a cycling time‐trial protocol. In contrast, a recent study has observed additive effects on muscular endurance when fatigue resistance is the primary limiting factor in resistance exercise (Montalvo‐Alonso et al. 2025). Our judo‐specific findings align more closely with the latter study, suggesting that whether co‐supplementation is beneficial may depend on task characteristics and the metabolic demands of the protocol.

A possible explanation for the compensatory interaction observed in this study is that the physiological benefits of BJ (i.e., improved perfusion, metabolic efficiency, and oxygenation) effectively counterbalanced the excessive metabolic cost induced by CAF during the early high‐intensity efforts. This compensatory effect is reflected in the prefinal SJFT physiological data. For example, plasma lactate in the BJCAF condition was elevated compared to PLA but lower than in CAF alone, while postexercise pH was higher than in CAF. The improved tissue oxygenation from NO‐mediated vasodilation may have enhanced the clearance of metabolic byproducts (e.g., H^+^, lactate) (Bailey et al. 2010) that accumulated during the early trials under CAF. This nitrate‐protective effect against acidosis likely explains why athletes in the BJ and BJCAF conditions maintained performance in the final trials, unlike those in the CAF condition. Furthermore, the cardiovascular data support this interplay; the maintained elevation of HR_0_ in the combined condition indicates preserved CAF‐induced stimulation. In contrast, the moderated HR_1_ response, lower than CAF alone in several SJFTs, suggests a regulatory cardiovascular effect of BJ. This balanced cardiovascular profile, coupled with enhanced muscle oxygen recovery kinetics, may have facilitated greater between‐trial recovery. This is evidenced by the sustained physical performance across all six SJFTs (Table 1) and the improved mean TT (Supporting Information S1: Figure S2).

Importantly, both the BJ and BJCAF conditions mitigated the development of cognitive fatigue in later trials. Nitrate supplementation has been shown to enhance cerebral blood flow and executive function, particularly under conditions of physiological stress and fatigue (Thompson et al. 2015; Wightman et al. 2015). The BJCAF exhibited the best overall cognitive performance (Supporting Information S1: Figures S6 and S7), suggesting that the bottom‐up physiological support provided by nitrate may sustain the top‐down arousal benefit of CAF, thereby helping to maintain vigilance during the later stages of the simulated competition day.

Metabolically, the marked elevation in plasma glucose with CAF reflects a classic catecholamine‐driven response that promotes glycogenolysis and hepatic glucose output (Laurent et al. 2000). While this may support acute energy availability, it is also a marker of significant physiological stress. In contrast, BJ supplementation resulted in a more moderate glucose response, consistent with its established role in improving metabolic efficiency rather than acutely elevating metabolic rate (Larsen et al. 2007). Additionally, the significant elevations in plasma nitrate/nitrite and CAF concentrations confirm protocol compliance and supplement bioavailability, providing a firm pharmacokinetic basis for the observed physiological and performance effects.

An additional assessment of practical significance was also performed using the smallest worthwhile change (SWC) and model‐based minimal detectable change at the 95% confidence level (MDC_95_) derived from within‐athlete variability (see Supporting Information S1: Supplementary Methods and Supporting Information S1: Table S1). All statistically significant between‐condition differences exceeded their respective SWC values, supporting the practical relevance of the observed effects. In particular, early‐stage performance advantages by CAF, the attenuation of performance decline with BJ, and the superior performance observed with BJCAF during the latter stages of the simulated competition day exceeded the corresponding SWC thresholds, indicating meaningful benefits from an applied performance perspective. Although none of the observed between‐condition effects exceeded the more conservative MDC_95_ threshold, the consistent pattern of SWC‐exceeding differences across multiple performance outcomes and trials suggests that the combined BJCAF strategy may provide practically meaningful advantages during repeated judo bouts performed across a competition day.

This study has several strengths. First, it simultaneously evaluated sport‐specific performance, neuromuscular function, cognitive performance, and a broad range of physiological and biochemical variables, providing a comprehensive assessment of the responses to supplementation. Second, unlike many previous studies that examined isolated exercise bouts, the present investigation employed a prolonged simulated competition day, thereby enhancing the practical relevance of the findings for combat sports. Third, the use of trained judo athletes, ecologically valid randori‐based exercise, and low habitual CAF consumers increased both sport specificity and sensitivity to supplementation effects. Finally, biochemical analyses confirmed supplement uptake and supported the interpretation of the observed physiological and performance responses.

This study also has several limitations. First, although we controlled for menstrual phase in female participants, hormonal fluctuations across the cycle can meaningfully affect substrate utilization and fatigue resistance; larger female cohorts with hormonal confirmation would be needed to examine these interactions. Second, our simulated competition day was intentionally rigorous, but it cannot completely replicate the psychological stress, opponent variability, and irregular recovery intervals of real tournaments. Likewise, participants were instructed to exert maximal effort on every trial for experimental control; this contrasts with real competition, where judo athletes commonly adopt tactical pacing and energy‐conservation strategies to preserve capacity for later decisive matches. Although the use of free randori enhanced the ecological validity of the protocol, we did not quantify combat structure variables such as the effort‐to‐pause ratio, attack frequency, or the distribution of effort intensities. Furthermore, repeated exposure to the same opponents across sessions may have influenced tactical behavior and pacing strategies despite matching opponents for body mass and skill level. Consequently, some between‐session variability in physiological and performance responses cannot be excluded. These design choices improve ecological validity but reduce experimental control over match‐related demands. Third, we provided a standardized isotonic beverage after each trial to control hydration and energy availability. While this increases ecological validity, supplying carbohydrate to all conditions could have blunted between‐condition metabolic differences and thus influenced some outcomes. Fourthly, only a single CAF dose (3 mg/kg) was investigated. Therefore, the present findings cannot be generalized to higher or lower CAF doses, and future studies should determine whether different dosing strategies influence performance responses during prolonged competition‐day simulations. Finally, mechanistic interpretations should be made with caution because we did not directly assess physiological processes that may underlie the observed performance responses, such as substrate utilization, neuromuscular activation, or molecular signaling pathways related to CAF and dietary nitrate supplementation. Consequently, the proposed mechanisms remain inferential and are based on systemic physiological responses and performance outcomes. Future studies incorporating additional mechanistic assessments, larger female cohorts, and competition formats that more closely replicate tournament demands would further improve our understanding of the factors mediating performance during prolonged judo competition.

## 5 Conclusion

This study demonstrates that the ergogenic effects of acute 3 mg/kg CAF supplementation were evident during the early stages of the simulated competition day but were not maintained during the later stages, when judo‐specific performance, neuromuscular function, and cognitive vigilance declined. In contrast, BJ produced a distinct late‐stage benefit, improving exercise efficiency and supporting cardiovascular recovery during later bouts. Combined CAF and BJ supplementation yielded the most favorable overall profile, attenuating the later‐session decline observed with CAF alone and supporting sustained high‐level performance across the competition day. These results highlight the need to consider temporal dynamics and metabolic costs when designing nutritional strategies for multi‐bout, high‐intensity sports tournaments such as judo.

From an applied perspective, these findings have important implications for judo athletes, and likely other combat‐sports athletes who must perform multiple high‐intensity matches during a single competition day. The present findings suggest that a single CAF supplementation strategy may not optimally support performance throughout a prolonged competition day. Coaches and sport nutritionists should therefore be aware that although CAF enhanced performance during the early stages of the protocol, this benefit was not sustained in later bouts. Accordingly, the timing of supplementation, dosing strategy, and potential co‐administration with complementary ergogenic aids should be considered when designing nutritional interventions for multi‐bout competitions. In contrast, BJ produced sustained benefits in later trials, indicating it may be particularly useful in formats requiring multiple successive matches; however, its modest early‐trial effects suggest it may not be sufficient if peak performance is required immediately. The combined BJCAF strategy delivered the most balanced profile in our simulation, providing early performance enhancement while attenuating late fatigue.

## Author Contributions

D.K., H.S. and S.K. designed the study. S.K., O.G., V.A., E.Ç. and S.A. collected the data. D.K., H.S. and S.K. analyzed and interpreted the data. S.K., O.G., V.A., E.Ç. and S.A. prepared the initial draft. D.K. and H.S. reviewed and edited the original draft. All authors have read and agreed to the final manuscript.

## Funding

The authors have nothing to report.

## Ethics Statement

The study was approved by the Ethics Committee of International Science and Technology University (202507‐01) and conducted in accordance with the Declaration of Helsinki. The trial was registered with the Iranian Registry of Clinical Trials (IRCT20231004059615N5).

## Conflicts of Interest

The authors declare no conflicts of interest.

## Supporting information


Supporting Information S1

## Data Availability

The data that support the findings of this study are available on request from the corresponding author. The data are not publicly available due to privacy or ethical restrictions.
