# Supplementary material for: The Effect of Acute Beetroot Juice and Caffeine Supplementation on Specific Judo Performance During a Simulated Competition Day in Judo Athletes
Source: Eur J Sport Sci. 2026 Oct 21;26(11):e70278. doi: 10.1002/ejsc.70278 (PMC13685822; doi:10.1002/ejsc.70278)
Supplement: Supplementary file 1 [file EJSC-26-e70278-s001.docx]

**Supplemental Material**

**Supplemental Methods**

**Smallest worthwhile change and Minimal detectable change calculation**

To evaluate the practical significance of treatment-induced changes, the smallest worthwhile change (SWC) and minimal detectable change (MDC) were calculated for the performance outcomes. Because the present study employed a repeated-measures crossover design involving athletes from different sexes and weight categories, SWC was derived from the within-athlete variability estimated using a linear mixed-effects model rather than from the overall between-subject standard deviation. Specifically, a mixed model including participant as a random intercept and time as a repeated factor with a first-order autoregressive [AR(1)] covariance structure was fitted to the placebo condition data. The within-athlete standard deviation (SD_within_) was obtained as the square root of the repeated-measure variance parameter. The SWC was subsequently calculated as 0.3 × SD_within_, consistent with recommendations for athlete monitoring and performance tracking, where within-subject variability provides a more meaningful benchmark than between-subject variability in heterogeneous athletic populations (1,3).

A model-based minimal detectable change at the 95% confidence level (MDC_95_) was also calculated to quantify the minimum change exceeding normal within-athlete variability. Because a separate test–retest reliability study was not conducted, MDC_95_ was estimated from the residual within-subject variability obtained from the mixed model. Specifically, it was calculated as MDC_95_ = 1.96 × √2 × SD_within_. This approach provides a conditional estimate of the magnitude of change required to exceed expected within-athlete variation under the measurement conditions of this study and has been recommended when repeated observations are available but classical test–retest estimates are not (2). Changes exceeding the SWC were considered practically meaningful, whereas changes exceeding the MDC_95_ were interpreted as likely to represent true changes beyond normal within-athlete variability.

**Table S1. The smallest worthwhile change (SWC), model-based** **minimal detectable change (MDC_95_), and meaningful intervention effects for performance outcomes**

| **Outcomes** | **SWC** | **Model-based MDC_95_** | **∆ > SWC** | **∆ > Model-based MDC_95_** |
| --- | --- | --- | --- | --- |
| **TT** (n) | 0.24 | 2.22 | BJ *vs.* PLA: SJFTs 2-4↑, 5-6↑^*^;  CAF *vs.* PLA: SJFTs 1-3↑^*^, 4↑, 5↓^*^, 6↓;  BJCAF *vs.* PLA: SJFTs 1-3↑^*^, 4↑, 5-6↑^*^;  CAF *vs.* BJ: SJFTs 1-2↑^*^, 3↑, 5-6↓^*^;  BJCAF *vs.* BJ: SJFTs 1-2↑^*^, 3↑, 6↓;  BJCAF *vs.* CAF: SJFTs 2↓, 4↑, 5-6↑^*^. | - |
| **SJFT index** | 0.16 | 1.45 | BJ *vs.* PLA: SJFTs 1↓,2↓, 3-6↓^*^;  CAF *vs.* PLA: SJFTs 1↓, 2↓^*^, 4↑, 5-6↑^*^;  BJCAF *vs.* PLA: SJFTs 1-3↓, 6↓;  CAF *vs.* BJ: SJFTs 3↑, 4-6↑^*^;  BJCAF *vs.* BJ: SJFTs 4↑, 5-6↑^*^;  BJCAF *vs.* CAF: SJFTs 3-4↓, 5-6↓^*^. | - |
| **Jump height** (cm) | 0.55 | 5.13 | BJ *vs.* PLA: Trials 3-5↑^*^;  CAF *vs.* PLA: PE↑^*^, Trials 1-3↑^*^, 5↓^*^;  BJCAF *vs.* PLA: PE↑^*^, Trials 1-5↑^*^;  CAF *vs.* BJ: PE↑^*^, Trials 1-3↑^*^, 4↓, 5↓^*^;  BJCAF *vs.* BJ: PE↑^*^, Trials 1-3↑^*^;  BJCAF *vs.* CAF: Trials 4↑, 5↑^*^. | - |
| **FT:CT ratio** | 0.006 | 0.06 | BJ *vs.* PLA: PE↑, Trials 1↑, 3↑, 4-5↑^*^;  CAF *vs.* PLA: PE↑^*^, Trials 1-3↑^*^, 4↑, 5↓^*^;  BJCAF *vs.* PLA: PE↑^*^, Trials 1-5↑^*^;  CAF *vs.* BJ: PE↑^*^, Trials 1-2↑^*^, 3↑, 4↓, 5↓^*^;  BJCAF *vs.* BJ: PE↑^*^, Trials 1-3↑^*^, 4-5↑;  BJCAF *vs.* CAF: Trials 3-4↑, 5↑^*^. | - |
| **HGS** (kg) | 0.31 | 2.9 | BJ *vs.* PLA: PE↑, Trials 2↑, 4↑, 5↑^*^;  CAF *vs.* PLA: PE↑^*^, Trials 1-2↑^*^, 3↑, 5↓;  BJCAF *vs.* PLA: PE↑^*^, Trials 1-2↑^*^, 3↑, 4-5↑^*^;  CAF *vs.* BJ: PE↑^*^, Trials 1-2↑^*^, 3↑, 4↓, 5↓^*^;  BJCAF *vs.* BJ: PE↑^*^, Trials 1-3↑^*^, 4-5↑;  BJCAF *vs.* CAF: Trials 1-2↓, 4-5↑^*^. | - |
| **Number of lapses** | 0.33 | 3.08 | BJ *vs.* PLA: Trials 4↓^*^, 5↓;  CAF *vs.* PLA: PE↓^*^, Trials 1-2↓^*^, 4↑, 5↑^*^;  BJCAF *vs.* PLA: PE↓^*^, Trials 1-2↓^*^, 3-5↓;  CAF *vs.* BJ: PE↓^*^, Trials 1-2↓^*^, 4-5↑^*^;  BJCAF *vs.* BJ: PE↓^*^, Trials 1-2↓^*^;  BJCAF *vs.* CAF: Trials 1↑, 4-5↓^*^; | - |
| **Mean response speed** (1/s) | 0.024 | 0.22 | BJ *vs.* PLA: Trials 1↑, 4↑, 5↑^*^;  CAF *vs.* PLA: PE↑^*^, Trials 1-3↑^*^, 5↓^*^;  BJCAF *vs.* PLA: PE↑^*^, Trials 1-2↑^*^, 3↑, 4↑^*^, 5↑;  CAF *vs.* BJ: PE↑^*^, Trials 2-3↑^*^, 4-5↓^*^;  BJCAF *vs.* BJ: PE↑^*^, Trials 1↑, 2↑^*^;  BJCAF *vs.* CAF: Trials 4-5↑^*^. | - |

SJFT, Special Judo Fitness Test; TT, total number of throws; FT:CT, flight-time:contraction-time; HGS, handgrip strength; PE, pre-exercise; *, statistically significant (p < 0.05).

**Figure S1.** Paired individual data with connecting lines for total number of throws (**A**), Special Judo Fitness Test index (**B**), jump height (**C**), flight-time/contraction-time ratio (**D**), hand grip strength (**E**), number of lapses (**F**), mean response speed (**G**), and tissue saturation index (**H**) to facilitate visualization of within-participant trajectories across the four supplementation conditions.

**Figure S2.** Effects of beetroot juice (BJ), caffeine (CAF), and beetroot juice + caffeine (BJCAF) supplementation on the mean values of total number of throws (TT; **A**), heart rate immediately at end of the test (HR_0_; **B**), heart rate 1 minute after the test (HR_1_; **C**), Special Judo Fitness Test (SJFT) index (**D**), and rate of perceived exertion (RPE; **E**) during each condition. There was a significant difference between the conditions for mean values of TT (F = 11.564, p < 0.001, ηp² = 0.25), HR_0_ (F = 35.772, p < 0.001, ηp² = 0.51), HR_1_ (F = 22.472, p < 0.001, ηp² = 0.4), and SJFT index (F = 15.596, p < 0.001, ηp² = 0.31), but not for RPE (F = 2.412, p = 0.106, ηp² = 0.06). ^a^ P < 0.05 significant difference from PLA; ^b^ P < 0.05 significant difference from BJ; ^c^ P < 0.05 significant difference from CAF; ^d^ P < 0.05 significant difference from BJCAF.

**Figure S3.** Effects of beetroot juice (BJ), caffeine (CAF), and beetroot juice + caffeine (BJCAF) supplementation on the percent changes of jump height in countermovement jump to pre-exercise (**A**), and mean values of jump height during each condition (**B**). There was a condition × time interaction effect (F = 8.746, p < 0.001, ηp² = 0.2) on the percent changes of jump height in CMJ to pre-exercise. The mean values of jump height also revealed differences between the conditions (F = 21.222, p < 0.001, ηp² = 0.38). ^a^ P < 0.05 significant difference from PLA; ^b^ P < 0.05 significant difference from BJ; ^c^ P < 0.05 significant difference from CAF; ^d^ P < 0.05 significant difference from BJCAF.

**Figure S4.** Effects of beetroot juice (BJ), caffeine (CAF), and beetroot juice + caffeine (BJCAF) supplementation on the percent changes of flight-time/contraction-time (FT:CT) ratio in countermovement jump (CMJ) to pre-exercise (**A**), and mean values of FT:CT ratio during each condition (**B**). There was a condition × time interaction effect (F = 18.488, p < 0.001, ηp² = 0.35) on the percent changes of FT:CT ratio in CMJ to pre-exercise. The mean values of FT:CT ratio also revealed differences between the conditions (F = 8.774, p < 0.001, ηp² = 0.2). ^a^ P < 0.05 significant difference from PLA; ^b^ P < 0.05 significant difference from BJ; ^d^ P < 0.05 significant difference from BJCAF.

**Figure S5.** Effects of beetroot juice (BJ), caffeine (CAF), and beetroot juice + caffeine (BJCAF) supplementation on the percent changes of the hand grip strength (HGS) to pre-exercise (**A**), and mean values of HGS during each condition (**B**). There was a condition × time interaction effect (F = 16.527, p < 0.001, ηp² = 0.33) on the percent changes of HGS to pre-exercise. The mean values of HGS also revealed differences between the conditions (F = 10.178, p < 0.001, ηp² = 0.23). ^a^ P < 0.05 significant difference from PLA; ^b^ P < 0.05 significant difference from BJ; ^c^ P < 0.05 significant difference from CAF; ^d^ P < 0.05 significant difference from BJCAF.

**Figure S6.** Effects of beetroot juice (BJ), caffeine (CAF), and beetroot juice + caffeine (BJCAF) supplementation on the percent changes of the number of lapses to pre-exercise (**A**), and mean number of lapses during each condition (**B**). There was a condition × time interaction effect (F = 2.766, p < 0.05, ηp² = 0.08) on the percent changes of the number of lapses to pre-exercise. The mean number of lapses also revealed differences between the conditions (F = 18.577, p < 0.001, ηp² = 0.35). ^a^ P < 0.01 significant difference from PLA; ^b^ P < 0.001 significant difference from BJ; ^c^ P < 0.01 significant difference from CAF.

**Figure S7.** Effects of beetroot juice (BJ), caffeine (CAF), and beetroot juice + caffeine (BJCAF) supplementation on the percent changes of the mean response speed to pre-exercise (**A**), and the mean response speed during each condition (**B**). There was a condition × time interaction effect (F = 7.959, p < 0.05, ηp² = 0.19) on the percent changes of the mean response speed to pre-exercise. The mean response speed also revealed differences between the conditions (F = 12.263, p < 0.001, ηp² = 0.27). ^a^ P < 0.05 significant difference from PLA; ^b^ P < 0.05 significant difference from BJ; ^c^ P < 0.05 significant difference from CAF.

**References**

1.	Bonetti DL, Hopkins WG. Variation in performance times of elite flat-water canoeists from race to race. *International Journal of Sports Physiology and Performance* 5: 210–217, 2010.

2.	Hopkins WG. Measures of reliability in sports medicine and science. *Sports medicine* 30: 1–15, 2000.

3.	Hopkins WG, Marshall SW, Batterham AM, Hanin J. Progressive statistics for studies in sports medicine and exercise science. *Medicine & Science in Sports & Exercise* 41: 3–12, 2009.
